# Effect of *trans* Fatty Acid Intake on LC-MS and NMR Plasma Profiles

**DOI:** 10.1371/journal.pone.0069589

**Published:** 2013-07-29

**Authors:** Gözde Gürdeniz, Daniela Rago, Nathalie Tommerup Bendsen, Francesco Savorani, Arne Astrup, Lars O. Dragsted

**Affiliations:** 1 Department of Nutrition, Exercise and Sports, Faculty of Science, University of Copenhagen, Frederiksberg C, Denmark; 2 Department of Food Science, Faculty of Science, University of Copenhagen, Frederiksberg C, Denmark; Governmental Technical Research Centre of Finland, Finland

## Abstract

**Background:**

The consumption of high levels of industrial *trans* fatty acids (TFA) has been related to cardiovascular disease, diabetes and sudden cardiac death but the causal mechanisms are not well known. In this study, NMR and LC-MS untargeted metabolomics has been used as an approach to explore the impact of TFA intake on plasma metabolites.

**Methodology/Principal Findings:**

In a double-blinded randomized controlled parallel-group study, 52 overweight postmenopausal women received either partially hydrogenated soybean oil, providing 15.7 g/day of TFA (*trans*18:1) or control oil with mainly oleic acid for 16 weeks. Subsequent to the intervention period, the subjects participated in a 12-week dietary weight loss program. Before and after the TFA intervention and after the weight loss programme, volunteers participated in an oral glucose tolerance test. PLSDA revealed elevated lipid profiles with TFA intake. NMR indicated up-regulated LDL cholesterol levels and unsaturation. LC-MS profiles demonstrated elevated levels of specific polyunsaturated (PUFA) long-chain phosphatidylcholines (PCs) and a sphingomyelin (SM) which were confirmed with a lipidomics based method. Plasma levels of these markers of TFA intake declined to their low baseline levels after the weight loss program for the TFA group and did not fluctuate for the control group. The marker levels were unaffected by OGTT.

**Conclusions/Significance:**

This study demonstrates that intake of TFA affects phospholipid metabolism. The preferential integration of *trans*18:1 into the sn-1 position of PCs, all containing PUFA in the sn-2 position, could be explained by a general up-regulation in the formation of long-chain PUFAs after TFA intake and/or by specific mobilisation of these fats into PCs. NMR supported these findings by revealing increased unsaturation of plasma lipids in the TFA group. These specific changes in membrane lipid species may be related to the mechanisms of TFA-induced disease but need further validation as risk markers.

**Trial registration:**

Registered at clinicaltrials.gov as NCT00655902

## Introduction

Industrially produced *trans* fatty acids (TFA) are formed by partial hydrogenation of vegetable oil that changes *cis* configuration of double bond(s) to *trans*, resulting in solid fat for use in margarines and shortenings, and for commercial cooking, and manufacturing processes. Partially hardened oils are appealing for food industry owing to their properties such as long shelf life, their stability during deep-frying and their semi-solidity. However, consumption of TFA in the human diet has been associated with an increased risk of developing cardiovascular disease [1], [2], diabetes [3], and sudden death from cardiac causes [4]. TFA has now been banned in a few countries, including Austria, Denmark, Hungary, Sweden, and Switzerland as well as in California and in the New York municipality in the USA. Denmark was the first country where the background level of TFA exposure was minimized because the industry after the ban in 2004 succeeded in removing these fats from more than 90% all marketed products. However, this is not the situation in many other countries and studies to further document and understand the causes of TFA mediated coronary heart disease (CHD) risk are therefore still needed. This risk has been linked to the impact of TFA on lipoprotein metabolism, inflammation, and endothelial function [5]. It has been well documented that TFA intake increases low-density lipoprotein (LDL) cholesterol, reduces high-density lipoprotein (HDL) cholesterol, and increases the risk of cardiovascular disease [6], [7]. Nevertheless, the incidence of CHD reported in prospective studies as a result of TFA exposure has been greater than that predicted by increased serum lipids or inflammation alone. Thus, the observed associations between TFA consumption and cardiovascular disease events cannot be explained only by changes in lipoprotein levels, triglycerides, apolipoprotein (Apo) B/ApoAI ratio and C-reactive protein [8], implying that the mechanisms behind the adverse effects of TFAs are not fully understood. TFA exposure has also been associated with a higher risk of fatal ischemic heart disease [9] and sudden cardiac death [10]. Although the potential mechanism between TFA and sudden cardiac death is unclear, some have suggested that TFA may modulate cardiac membrane ion channel function [11] or have proarrhythmic properties, affecting cardiovascular electrophysiology [2].

Some evidence also pointed to a possible effect of TFA on obesity. A dietary 16-week intervention study was conducted here in 2008 by Bendsen et al. [13] to examine the effect of a high intake of industrially produced TFA (*trans*18:1) compared to the *cis* analog (*cis*18:1) on central obesity and insulin sensitivity. The low TFA background in Denmark made it feasible to conduct a TFA intervention against a clean background in the control group to unambiguously assess any shorter-term effects on central fat deposition or other risk markers of CHD. However, the study did not provide evidence for effects on obesity development. In order to search for new hypotheses and fill the gap between TFA intake and its detrimental health impacts, we selected an untargeted metabolomics approach to profile plasma samples from this study.

Metabolic profiling allows semiquantification of hundreds of metabolites in blood samples and might provide a unique insight into the potential underlying mechanisms. Many studies have demonstrated metabolomics as a powerful tool to understand responses of individuals with respect to their gene expression or alterations in their lifestyles and diets [12]. The application of liquid chromatography mass spectrometry (LC-MS) and nuclear magnetic resonance (NMR) in metabolomics for measurement of a wide range of metabolites in various biofluids has been well established. NMR provides high reproducibility and is a powerful tool in terms of quantification, whereas LC-MS is more sensitive, allowing detection of a larger number of chemical compounds, albeit with lower reproducibility.

Our results from the untargeted metabolomics analysis of the TFA intervention study revealed an increased presence during TFA intake of membrane-derived, specific long chain polyunsaturated fatty acid (PUFA)-containing PCs and a SM, suggesting the possibility of using these compounds as individual markers of TFA integration into plasma membranes.

## Materials and Methods

### Subjects

A total of 64 volunteers were assessed for eligibility and 52 healthy, moderately overweight (body mass index between 25 and 32 kg m^−2^), normolipidemic (LDL-C below 6 mmol/L) postmenopausal women, between 45 to 70 years of age, were recruited in this study. Detailed description of participant recruitment and enrolment, inclusion and exclusion criteria, randomization and compliance has been published previously [13]. CONSORT flow diagram displaying subjects recruited into the dietary intervention has been in given Figure S1.

### Study Design

The dietary intervention study had a randomized, double-blind, parallel design. The clinical trial protocol is given as supporting information (Protocol S1). The subjects were randomized by strata of waist circumference with a cut-off at 96 cm to assure that both strata were equally represented in the two groups. Randomization numbers from a pre-defined list were provided by a third party to the kitchen who delivered bread rolls differing in their TFA content to the subjects. Subjects were given 26 g/d of partially hydrogenated soybean oil with approximately 60% *trans* fats (TFA group; n = 25) or 50/50% mix of palm oil and high oleic sunflower oil as the control oil (CTR group; n = 27) for 16 weeks. Both test oils were supplied by Aarhus Karlshamn, Aarhus C, Denmark. The fatty acid composition in the oils has been described elsewhere [13]. Briefly, the two fats differed in the content of TFA (18:1 *trans*-9, 18:1 *trans*-8, 18:1 *trans*-7), palmitic (16:0), oleic (18:1 *cis*-9) and linoleic acids (18:2 *cis*-6). The fats were incorporated into bread rolls providing a total of 600 kcal/d (41 E% from fat), equivalent to 28% of the subjects' energy requirements on average. Frozen rolls were handed out to the subjects every 1–4 weeks from the department for consumption at home.

The women visited the department for four examinations during the intervention: at screening (1–8 weeks prior to baseline), baseline (w0), mid-intervention (week 8) and at the end of treatment (w16). In addition, the subjects attended the department for control weighing at weeks 4 and 12. Subjects were instructed to maintain their habitual activity level throughout the dietary intervention period. Subsequent to the dietary intervention period, the subjects participated in a 12-week (w28) dietary weight loss program. The blood samples for metabolomics analysis were collected only at w0, w16 and w28.

Dietary intake was measured using 3-day weighed food records at baseline and in the last week of the intervention. The only significant dietary differences between diet groups during the intervention were the contributions of energy from monounsaturated cis-fatty acids (MUFA) and TFA, indicating that the diets were overall comparable apart from the *cis/trans* fatty acid composition. The intake of TFA was higher (7.0±0.2 E% [mean ± SEM] vs. 0.3±0.0 E%) and the intake of cis-MUFA was lower (10.3±0.4 E% vs. 13.4±0.8 E%) in the TFA group compared with the CTR group [13]. The trial was registered at clinicaltrials.gov as NCT00655902.

### Ethics statement

The subjects were given both verbal and written information, whereupon all gave written consent. The study was carried out at the Department of Nutrition, Exercise and Sport, University of Copenhagen, Frederiksberg, Denmark, between April 2008 and March 2009 and was approved by the Municipal Ethical Committee of The Capital Region of Denmark in accordance with the Helsinki-II declaration (H-B_2007-089). A copy of the document issued by ethical committee, confirming your study was considered and approved was included as supporting information (Document S1). Subjects received 900 US$ as compensation on completion of all the tests. This level of compensation is customary in Denmark for participation in longer-term demanding trials to cover transportation, discomfort, and the time spent but is taxed at a level of approximately 50%. The overweight volunteers were further offered a free 3-months weight loss programme. With the weight loss programme we aimed to reverse the putatively unbeneficial effect of the *trans*-fat, and thereby we anticipated that the subjects would be healthier after than before participation in the study.

### Blood sampling

Prior to each visit, the subjects were told to fast for at least 10 hours (except for 0.5 L water). They were instructed to avoid alcohol consumption and vigorous exercise on the day before and to consume similar carbohydrate-rich evening meals on the evening before each visit. Body weight and height were measured by standard procedures.

Insulin sensitivity was assessed by use of frequent sampling 3-hour oral glucose tolerance tests (OGTTs) where subjects ingested a solution of 75 g glucose dissolved in 300 mL water. Venous blood samples were collected before and during the OGTT at −10, 30 and 120 minutes into 4 mL EDTA-coated tubes (BD Medical, Albertslund, Denmark). The blood was centrifuged at 3000 g at 4°C for 10 min. The plasma fraction was portioned into 2 mL cryotubes (Nunc, Roskilde, Denmark) and stored at −80°C until further processing.

### Chemicals

Authentic standards of PC(18:0/18:2), PC(*cis*18:1/*cis*18:1), *trans* PC(*trans*18:1/*trans*18:1), PC(18:0/18:2), PC(18:0/20:4), PC(18:0/22:6), PC(17:0/0:0), PC(17:0/17:0), PE(17:0/17:0), PG(17:0/17:0), Cer(d18:1/17:0), PS(17:0/17:0), PA(17:0/17:0) were purchased from Avanti Polar Lipids Inc. (Alabaster, AL, USA). Standards of racemic MG(17:0/0:0/0:0), racemic DG(17:0/17:0/0:0), TG(17:0/17:0/17:0), PC(16:1/0:0-D3), PC(16:1/16:1-D6), and TG(16:0/16:0/16:0-13C3) were purchased from Larodan Fine Chemicals AB (Malmö, Sweden).

### LC-QTOF-MS analysis

Plasma protein precipitation was performed, as described earlier [14]. An ultra-performance liquid chromatography (UPLC) system coupled to quadruple time-of-flight (Premier QTOF) mass spectrometer (Waters Corporation, Manchester, UK) was used for sample analysis. The mobile phase was 0.1% formic acid in water (A) and 0.1% formic acid in 70% acetonitrile and 30% methanol (B). Five µL of each sample were injected into a HSS T3 C_18_ column (2.1×100 mm, 1.8 µm) coupled with a VanGuard HSS T3 C_18_ column (2.1×5 mm, 1.8 µm) operated for 7.0 min. The eluate was analyzed by electrospray ionization (ESI)-QTOF-MS (Premium QTOF, Waters) in positive and negative mode, applying a capillary voltage of 3.2 kV and 2.8 kV, respectively and cone voltage of 20 kV. Ion source and desolvation gas (nitrogen) temperatures were set at 120 and 400°C, respectively. More detailed UPLC-QTOF analysis conditions were explained previously [15]. Blanks (5% of acetonitrile∶methanol 70∶30 v/v in water) and external metabolomics standard mixtures were injected every 30 plasma samples throughout each analytical batch.

In order to identify relevant metabolites, MS/MS fragmentation analyses were performed by post-column infusion experiments conducted as follows: a 1.6 mM solution of lithium formate dissolved in water-propanol (1∶1) was infused at 4 uL/min using a Waters built-in syringe pump. Both flows, from the UPLC column and the infusion pump, were combined using a zero-dead-volume ‘T’ union and introduced into the mass spectrometer. The MS/MS experiment was conducted in positive ion mode operating in product ion scan. The collision-induced dissociation (CID) energy was set at 25 eV and the MS/MS scan range at m/z 100-850. All other parameters were set to the same values with the MS experiment.

In order to verify the findings of lipophilic markers, we performed a lipidomics analysis of 12 samples from each treatment group at baseline and at the end of the intervention. Each sample was added with the internal standards, PC(17:0/0:0), PC(17:0/17:0), PE (17:0/17:0), PG(17:0/17:0), Cer(d18:1/17:0), PS(17:0/17:0), PA(17:0/17:0), racemic MG(17:0/0:0/0:0), racemic DG(17:0/17:0/0:0) and TG(17:0/17:0/17:0) . The concentration of each standard was approximately 0.1 µg/sample. The samples were extracted as described previously [14], but an additional extraction with 200 uL chloroform∶methanol (2∶1 v/v) was performed on the Sirocco^R^ filter support by gentle shaking with the precipitated protein for 5 min followed by opening of the valves to collect the additional extract. The combined extract was evaporated to dryness and redissolved in 190 uL water-saturated chloroform-methanol (2∶1). Before injection 0.1 µg of the following additional standards were added in 10 µL of the same solvent: PC(16:1/0:0-D3), PC(16:1/16:1-D6), and TG(16:0/16:0/16:0-13C3), as described by Nygren et al (2011). The samples were injected on the UPLC-QTOF system using a HSS T3 C_18_ column (2.1×100 mm, 1.8 µm) coupled with a VanGuard HSS T3 C_18_ column (2.1×5 mm, 1.8 µm). Solvent A was 1% 1 M NH_4_Ac and 0.1% HCOOH in water and solvent B was acetonitrile:2-propanol (1∶1, v/v), 1% 1 M NH4Ac and 0.1% HCOOH. A 6 min gradient from 100% A to 100% B was used. A gradient in flow was also applied starting from 0.2 mL/min, increasing to 0.5 mL/min over 3 min and going back to starting conditions at 10 min with 2 min re-equilibration time before the next injection.

#### Identification of lipids

**Figure 1:**
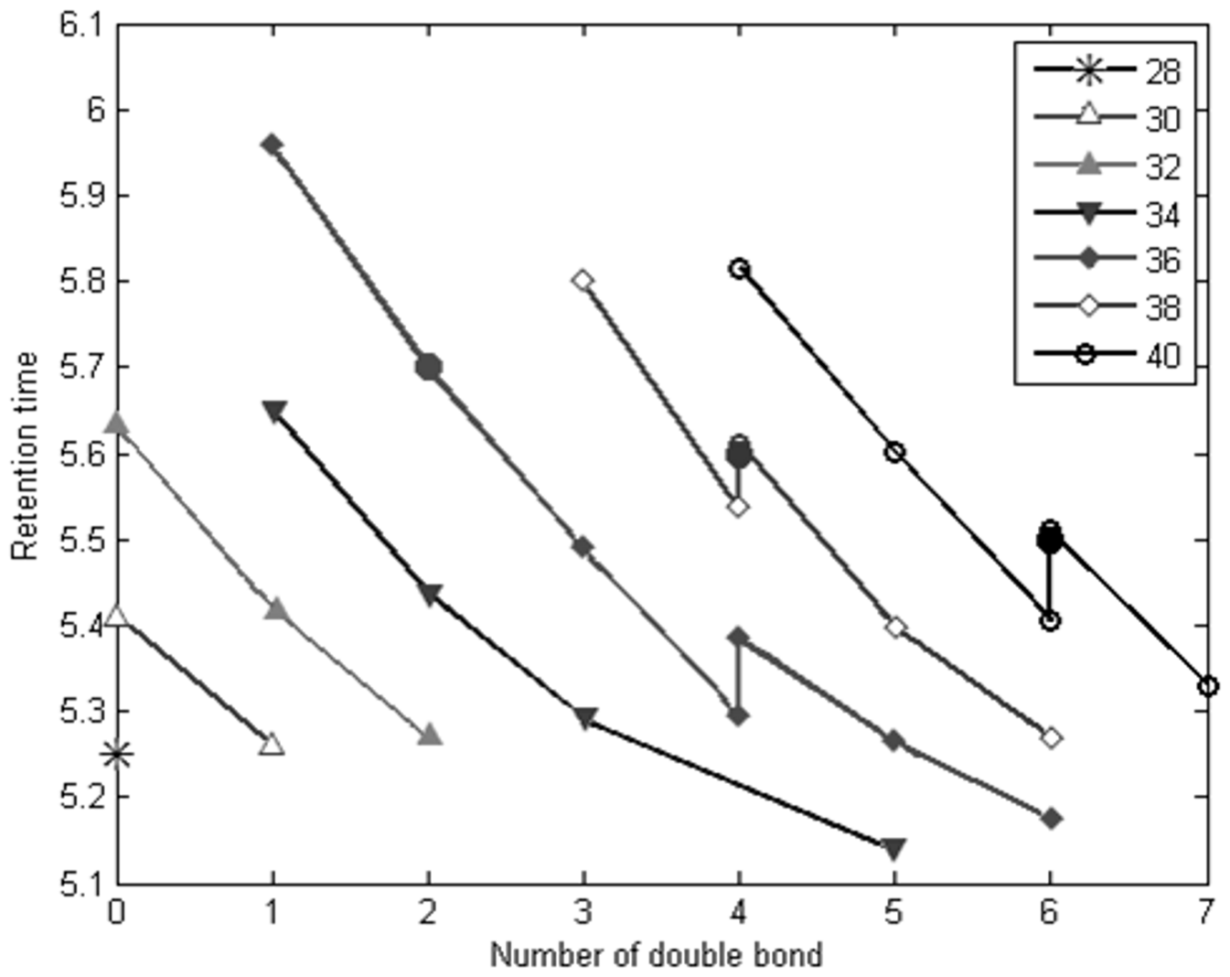
The observed retention time values of identified PCs (empty circles). Filled circles illustrate retention time of the authentic standards, PC(18:1/18:1), PC(18:0/20:4) and PC(18:0/22:6), confirming the predicted pattern.

Authentic standards PC(18:0/18:2), PC(*cis*18:1/*cis*18:1), PC(*trans*18:1/*trans*18:1), PC(18:0/18:2), PC(18:0/20:4), and PC(18:0/22:6) were analysed by LC-MS with sample analysis instrumental conditions. As it was not possible to purchase the standard compound for each PC and SM, we developed a simple algorithm to identify the various PCs and SMs by their retention time and m/z. An increased number of carbon atoms results in decreased polarity and increased retention time. In addition, for a PC, SM or lysophosphatidylcholine (LPC) with a specific carbon number, an increasing number of double bonds in the fatty acyl chain reduces the retention time. Since each of the lipid species appear with its Na^+^ adduct, this information is utilized to remove irrelevant matches for positive mode data. The samples were analysed two years prior to the authentic standards which resulted in +0.1 min linear shift in retention time. Thus, 0.1 min was added to the retention time of each compound in the data set. As shown in Figure 1 for PCs, the retention times of authentic standards were matching almost precisely with the predicted ones (+0.1 min), thereby validating the model. Equally good matching was observed for retention times of authentic standard of SM (36:2) and the observed SM (36:2) (not shown). A few PCs appeared as two isomers, illustrated in Figure 1, corresponding to structural differences.

The structural characterization of compounds reflecting TFA intake was performed by their parent mass information and characteristic fragments in the CID spectrum of their lithated ions. PC(18:1/20:3) and PC(18:1/22:5) are identified with two orthogonal data; retention time and spectral information. PC(18:1/22:6) is annotated by the algorithm whereas SM(18:1/18:2) was characterised by MS/MS analysis using Li infusion. Further information about spectral fragmentation patterns (MS/MS) of the PC(18:1/20:3), PC(18:1/22:5), PC(18:1/22:6) and SM(18:1/18:2) is explained in detail as follows.

The product ion spectrum of lithated [M+Li]+ ions for PC(18:0/22:6) and PC(18:0/20:4) standards were comparable with the product ion spectrum of lithated [M+Li]+ ions in samples. A few PCs appeared as two isomers, illustrated in Figure 1, corresponding to structural differences of naturally occurring isomers. The ions arising from loss of trimethylamine [M-59], ethylene phosphate [M-183] and lithium ethylene phosphate [M-189] were common fragments for CID spectra of PCs and SMs. The earlier eluting isomer of PC(38:4) gave rise to the fragment ions 504.3, 528.3 and 534.3, matching with the potential fragmentation pattern of PC(18:1/20:3). The ions 504.3 and 528.3 represents the neutral loss of the sn-2 fatty acyl substituent as a lithium salt [M+Li-R2CO2Li]+ and as a ketene [M+Li-R2′CHCO]+, respectively [25], [26]. Moreover, the loss of sn-1 fatty acyl as a free fatty acid yielded the ion 534.3 corresponding to ([LPC(20:3)-H2O+Li]+). The later eluting isomer of PC(38:4) coeluted with our standard, PC(18:0/20:4). MS/MS spectra of the earlier eluting isomer of PC(40:6) implied contribution of two species (PC(18:1/22:5) and PC(20:2/20:4)) to a single chromatographic peak. The ions 552.3 and 526.3 resulted from loss of the sn-1 acyl group as a lithium salt from PC(18:1/22:5) and PC(20:2/20:4), respectively. The most abundant fragment was arising from the removal of the sn-1 substituent as a ketene. The later eluting isomer of PC(40:6) coeluted with our standard, PC(18:0/22:6). MS/MS fragmentation of PC(40:7) lead to its identification as PC(18:1/22:6) based on the fragment ions 550.3 ([M+Li-R1′CHCO]+) and 556.3 ([M+Li-R1CO2Li]+). MS/MS fragmentation of the pseudomolcular ion of PC(40:7) [M+H] on a Waters Synapt supported its identity with fragments 445.3 ([LPC(18:1)-OH]+), 504.3 ([M+H-R1CO2H] +), 568.3([M+H-R1′CHCO]+) and 522.3 ([M+H-R2′CHCO]+). However, the CID spectrum of SM(36:3) did not reveal any abundant ions that identify the fatty acyl substituents.

We putatively characterized PC(44:9), which is observed only as its potassium adduct, and we could therefore not include it into our prediction model in Figure 1. However, extrapolation of the model agrees with the observed retention time, 5.34 min.

### ^1^H NMR Analysis

Plasma samples were slowly thawed overnight at 4°C. Samples where then centrifuged 20 min at 12 k RPM and 300 µl plasma were transferred into a 5 mm NMR tube together with 300 µl of phosphate buffer at pH 7.4 containing at least 10% w/w D_2_O and gently mixed in order to avoid formation of bubbles/foam. 1D NOESY ^1^H NMR spectra were acquired on a Bruker DRX spectrometer (Bruker Biospin Gmbh, Rheinstetten, Germany) operating at 600,00 MHz for protons (14.09 Tesla) using a TCI cryo-probe head and equipped with a SampleJet autosampler. All samples were individually and automatically tuned, matched and shimmed. FIDs were Fourier transformed using a 0.3 Hz line broadening. The resulting spectra were automatically phased and baseline corrected using Topspin™ (Bruker Biospin), and the ppm scale was referenced towards the TSP peak at 0.00 ppm [16]. Assignment of resonances was done by comparison to literature values [17].

### Data Preprocessing

#### LC-MS

The raw data was converted to an intermediate netCDF format with the DataBridge™ utility provided with the MassLynx software. MZmine 2.7 [18] was employed for data preprocessing including the following steps: mass detection, chromatogram builder, chromatogram deconvolution (local minimum search), isotopic peaks grouper, peak alignment (join aligner) and gap filling. The final outcome from MZmine is a feature set where each feature is denoted by the mass over charge (m/z) ratio and a retention time.

MZmine preprocessed data was imported to MATLAB (Version 7.2, The Mathworks, Inc., MA, US). Peak filtering was applied based on two criteria. First, if a feature has a reasonable peak area (>60) in the first run blank sample, it is removed. Second, if a feature has a peak area lower than 5 (considered as noise level or gap filling errors), in more than 60% of the samples within both sample groups (TFA vs. CTR, in this case), it is excluded (percent rule, [19]).

To remove intra-individual variation, each feature is normalized with the mean of the two recordings (before and after intervention) for each subject at each OGTT time point (−10, 30 or 120 min) [19].

#### ^1^H NMR

The spectral alignment was performed by the icoshift algorithm [20]. Only the spectral region between 8.5 and 0.2 ppm was considered, and the spectral region containing the residual resonance from water (4.7–5.1 ppm) was removed. The spectral data set was normalized by using probabilistic quotient normalization [21] and reduced by an in-house implementation of the adaptative intelligent binning algorithm [22]. Varying bin size, within the boundaries of minimum 0.002 to a maximum 0.02 ppm, was used, depending on the peak width.

#### Data Analysis

The PLS_Toolbox (version 6.5, Eigenvector Research, Inc., MA, US) was used to implement the data analysis. Initially, principal component analysis (PCA) was applied to visualize grouping patterns and detection of outliers as an unsupervised multivariate data analysis method. Then, data was subjected to partial least squares-discriminant analysis (PLSDA) for classification purposes. PLSDA attempts to separate two groups of samples by regressing on a so-called dummy y-vector consisting of zeros and ones in the PLS decomposition. Permutation test [23] was applied with 1000 random assignments of classes. The test set sample classification errors were evaluated to qualify the classification results. Selectivity ratio [24], which provides a simple numerical assessment of the usefulness of each variable in a regression model, was chosen as the criteria for variable selection. Briefly, using the y-vector as a target, PLS components (in many cases more than one) are transformed into a single target-projected component. The variance explained by the target component is calculated for each variable and compared with the residual variance for the same variable. The ratio between explained and residual variance, called the selectivity ratio, represents a measure of the ability of a variable to discriminate different groups [24].

**Figure 2:**
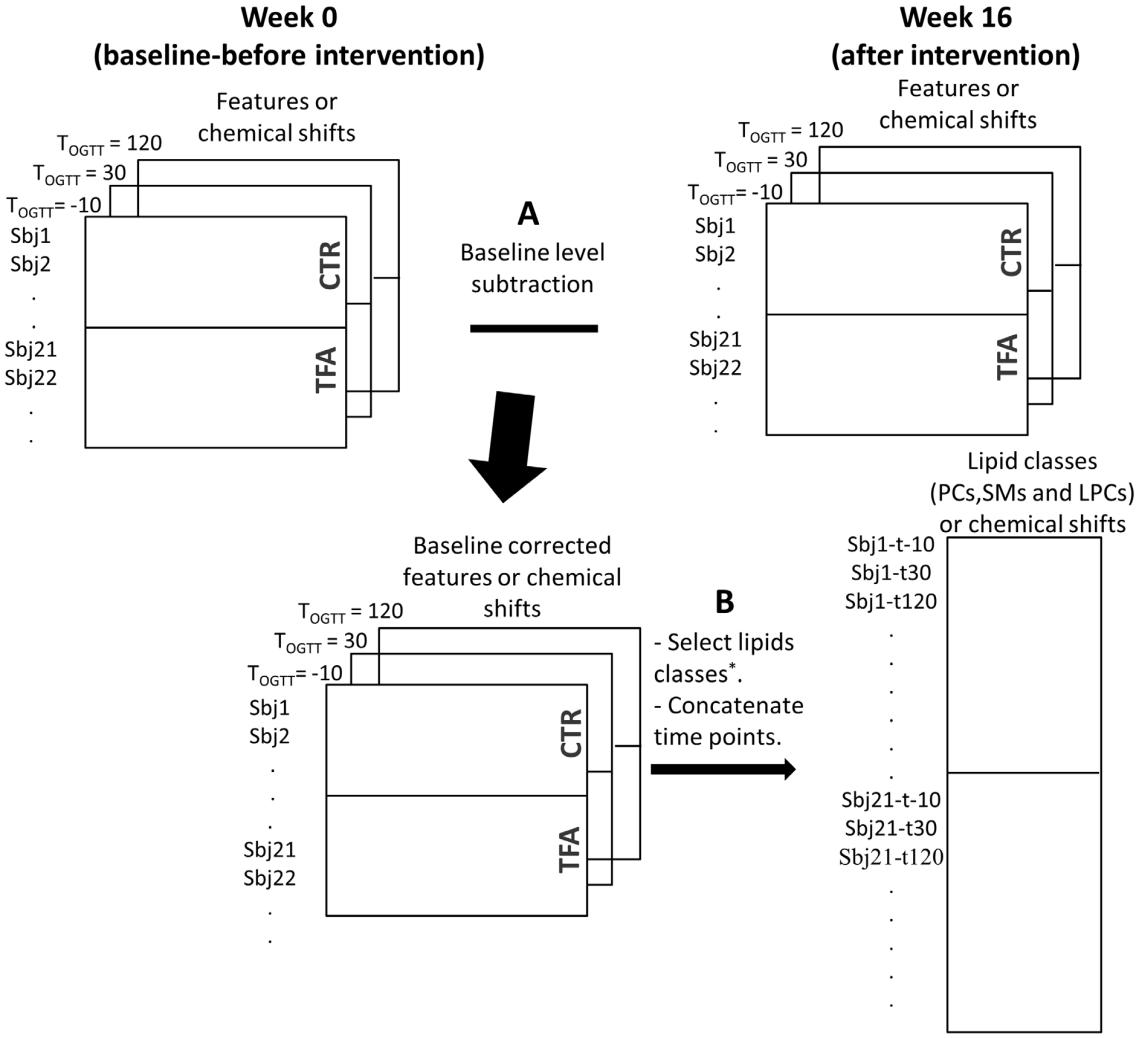
Data structure and arrangement scheme. Baseline subtraction (A) concatenation of time points (applied on LC-MS and NMR profiles), and selection of lipid classes (B) (applied on LC/MS data).

Data analysis was performed on baseline adjusted metabolite levels after intervention (w16-w0). Figure 2A illustrates data structure and the baseline adjustment scheme.

## Results

52 eligible overweight women were recruited for this study. A total of 49 participants completed the study, 24 out of 27 TFA-treated and 25 out of 25 controls. The test diets provided on average 28% of the subject's energy requirements. Self-reported compliance assessed using study diaries showed that 98% of all test bread rolls were consumed, with no difference between diet groups. The presence of elevated *trans*18:1 residues in RBC phospholipids determined by a gas chromatography/flame ionization detection was used as an objective compliance measure [13]. All subjects in the TFA group had elevated *trans*18:1 residue levels at both 8 and 16 weeks of intervention, whereas the control subjects did not (data published in [13]).

### Plasma ^1^H NMR profiles – extraction of TFA related patterns

Due to low sample amounts available, 42 NMR spectra were excluded, leaving 327 spectra (158 for TFA group, 169 for CTR group) for further analysis. Subsequent to binning, the spectral data set was condensed into 1493 binned ppm regions.

**Figure 3:**
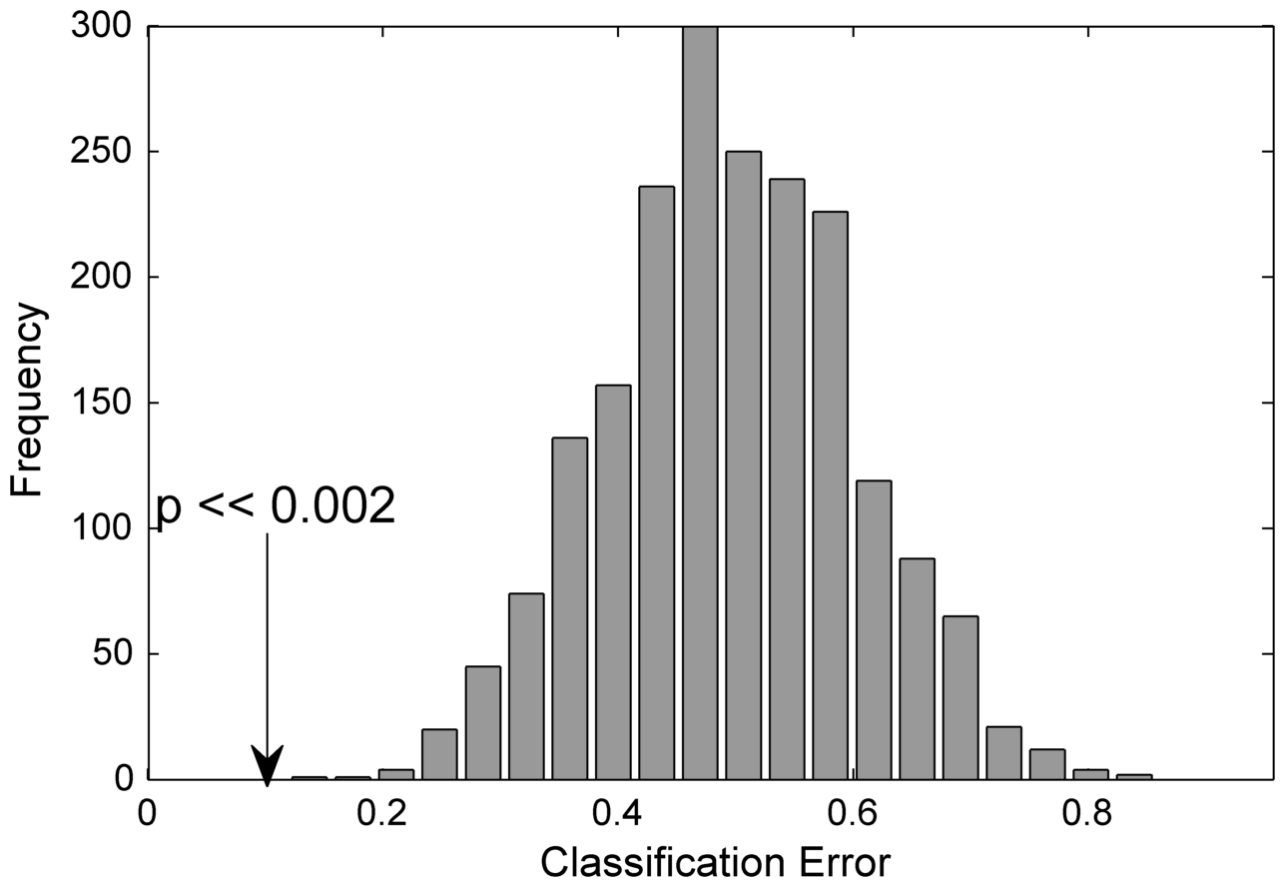
Permutation test results for NMR profiles. Class prediction results for NMR profiles based on test set predictions of the original labelling compared to the permuted data. P-values were calculated based on the comparison of classification error of the original model against the permutations.

PLSDA was applied individually for the data (baseline adjusted: w16-w0) including only one OGTT time point with the aim of discriminating CTR and TFA groups. The original classifications errors were barely significantly lower than the permuted ones (not shown). The classification performance was improved when we concatenated OGTT time points in the sample direction. The original and permuted data classification errors are given in Figure 3, none of the permutations had lower classification errors than the original ones.

The resonances reflecting TFA intake were selected based on evaluation of selectivity ratios from the PLSDA model (i.e. the resonances that have high selectivity ratio are more influential in discriminating between TFA and CTR groups). Annotation of discriminative resonances revealed elevated unsaturated lipids (δ 5.3) and LDL & VLDL (δ 1.28), methylenic protons for the TFA group, and an unassigned quartet (δ 3.23) for the CTR group.

Later, we included the measurements at w28 (i.e. 12 weeks after the end of the intervention). As mentioned earlier in this period all subjects had been under a weight loss program where the aim was to reverse the unbeneficial effects of TFA intake. Unlike PLSDA models on baseline corrected values (w12-w0), revealed no difference between TFA and CTR groups (classification error = 0.5). This demonstrates that the observed effects of TFA intake has been disappeared after weight loss program.

### Plasma LC-MS profiles – extraction of TFA related patterns

Inspection of LC-MS plasma profiles revealed that for 29 samples, many peaks were not apparent and other peaks had very low intensity. In all, 59 and 60 samples measurements remained for the TFA and CTR groups, respectively. A total of 2260 features in ESI positive mode and 1689 in ESI negative mode were detected by MZmine. After exclusion of noise and irrelevant features, by using blank samples and the percent rule, 767 and 710 features for positive and negative modes, respectively, remained for data analysis.

**Figure 4:**
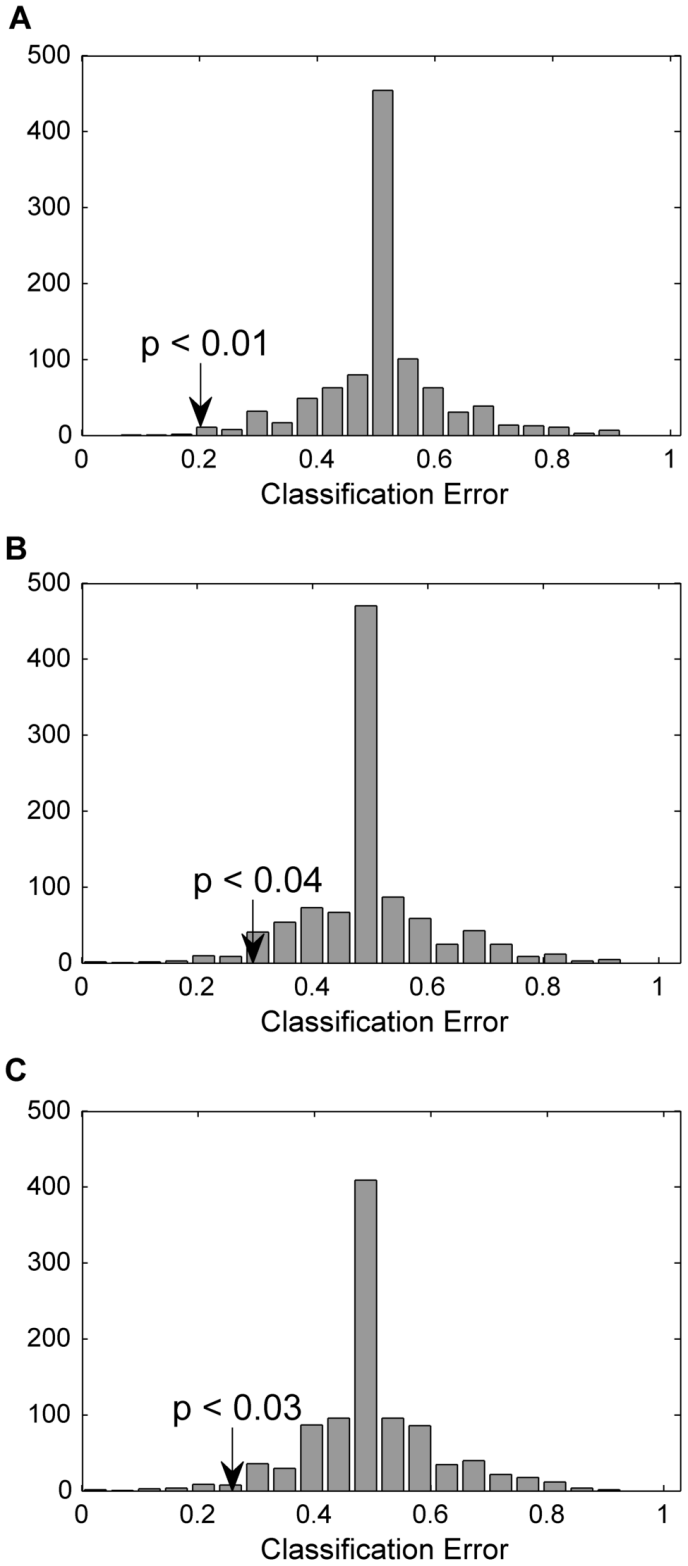
Permutation test results for LC-MS profiles at each OGTT time point. Class prediction results for LC-MS profiles based on test set predictions of the original labelling compared to the permuted data assessed using the classification errors. T_OGTT_ = −10 (A) T_OGTT_ = 30 (B) T_OGTT_ = 120 (C).

Initially, each OGTT time point was analysed individually by PLSDA with the aim of discriminating CTR and TFA groups. Permutation tests were applied to investigate potential PLSDA over-fitting issues. Classification error distributions from models with 1000 times permuted class identifiers together with the original classification error are presented in Figure 4. In case there were no differences between the groups, the expected classification error would be 0.5. Figure 4 perfectly matches this requirement. The comparison of classification error of the original model against the permutations was evaluated on the basis of p-values. Original classification errors were significantly lower than the permutations with p-values of 0.01 for T_OGTT_ = −10, 0.04 for T_OGTT_ = 30, and 0.03 for T_OGTT_ = 120 (α = 0.05).

Variable selection was performed based on the selectivity ratio from the PLSDA model using datasets from each OGTT time point. Features with the highest selectivity ratio were extracted (Table 1). Many of the discriminating features were common for the three OGTT time points indicating that TFA related patterns were not affected by OGTT. Identification of these features (as described in Materials and Methods section) pointed out that they were compounds from the lipid classes, PCs and SMs.

**Table 1:** Features with the highest selectivity ratio based on PLSDA models.

Measured m/z	Retention time* (min)	Suggested Compound	Suggested Adduct	Monoisotopic mass	Rank T_OGTT_ = −10	Rank T_OGTT_ = 30	Rank T_OGTT_ = 120
749.5614	5.31	SM(36:3)	[M+Na]^+^	726.5676	1	1	4
727.5781	5.31	SM(36:3)	[M+H]^+^	726.5676	2	4	7
832.5887	5.33	PC(18:1/22:6)	[M+H]^+^	831.5778	3	18	6
810.6072	5.52	PC(18:1/20:3)	[M+H]^+^	809.5934	4	9	25
854.5728	5.33	PC(18:1/22:5)	[M+Na]^+^	831.5778	5	2	1
922.5617	5.34	PC(44:9)	[M+K]^+^	883.6091	12	3	2
856.5728	5.41	PC(18:1/22:5)	[M+Na]^+^	833.5934	6	5	5

* 0.1 min was added to the retention time of each compound.

The importance of each feature was represented by its rank. The rank is based on each features sorted selectivity ratio in descending order.

A similar variable selection procedure was applied for negative mode, though PLSDA classification performance was lower compared to positive mode. Still, identical PC species (Table 1) were associated with TFA intake (data not shown). However, SM(36:3) was not detected in the negative mode which could be a potential reason for the lower classification performance.

**Figure 5:**
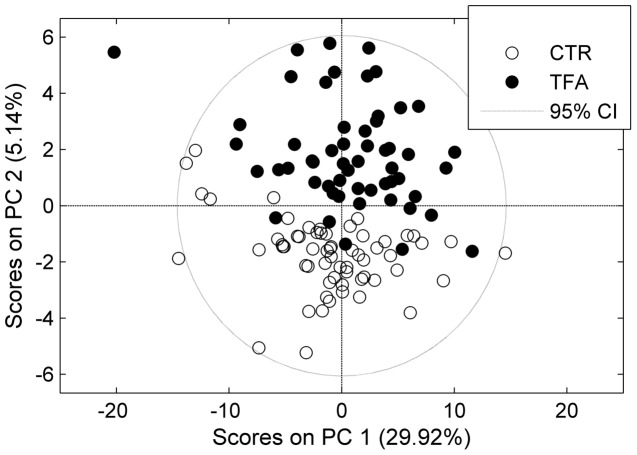
PC1 vs. PC2 scores plot of LC-MS based lipid profiles. The LC-MS profiles with concatenated time points including only LPCs, PCs and SMs as variables. Filled circles: TFA, empty circles: CTR.

**Figure 6:**
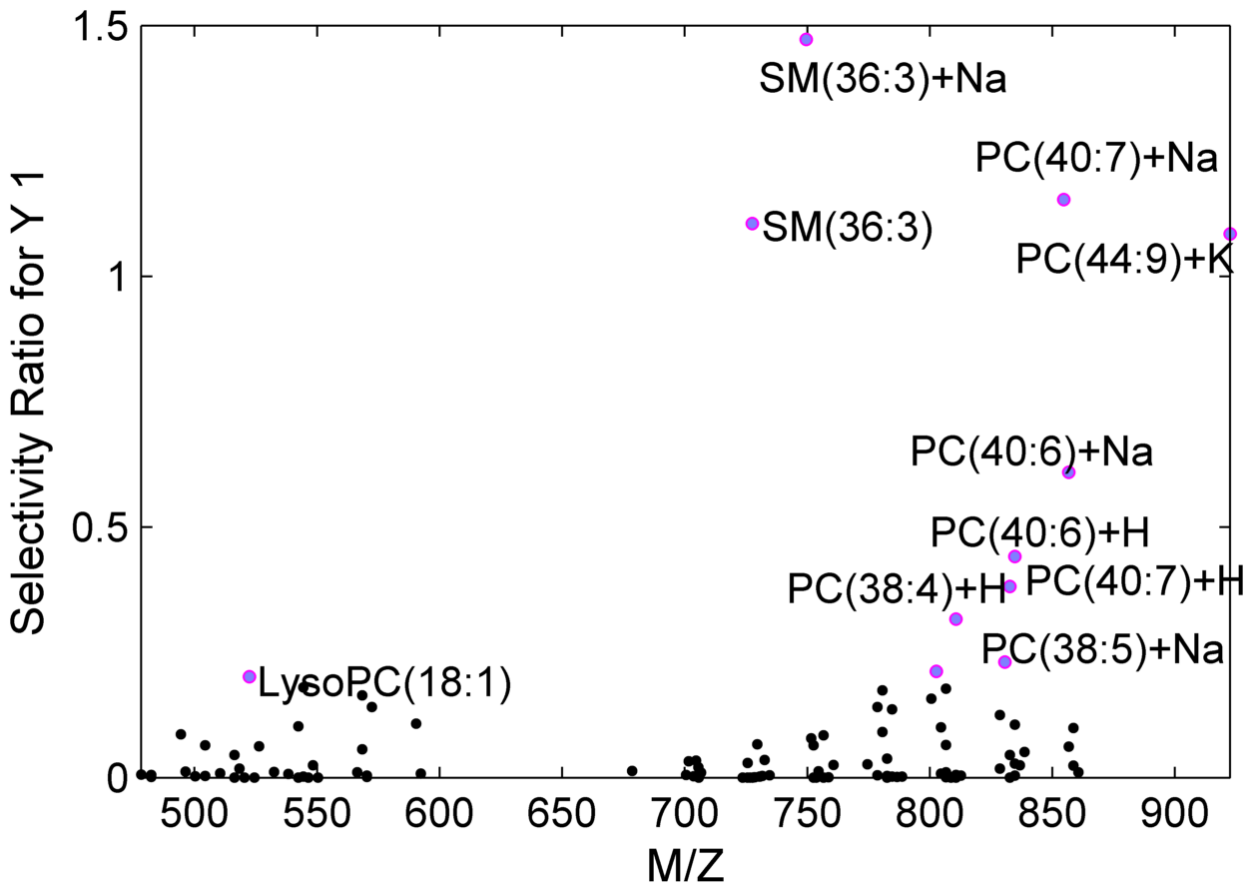
Selectivity ratio of each lipid species from the PLSDA model.

Since metabolites responding to the TFA exposure did not seem to be affected by OGTT, we concatenated the time points into a new data set, to increase the power of the classification model with a larger number of samples. In this case each subject was represented by three time points from OGTT measurements as illustrated in Figure 2B. Furthermore, as we have already demonstrated that only lipids were associated with TFA intake, features from the lipid classes (PC, SM and LPC) were included as variables (Figure 2B). The idea behind targeting the lipids was to explore whether only the specific PCs and the SM mentioned in Table 1 respond to TFA intake or if there are other relevant lipids that could be blurred due to the large number of variables. The PCA scores plot is shown in Figure 5. The control group clearly separated from the TFA group in the second principal component. Samples from different time points were quite spread in both CTR and TFA clusters and none of the principal components explained OGTT (not shown). Later, PLSDA was applied to select the main contributing lipids. The classification errors, sensitivity and specificity of cross validated samples were 0.04, 0.85 and 0.88, respectively. The calculated selectivity ratios were the largest for the lipid compounds given in Table 1 (Figure 6) which were all increased with TFA intake.

**Figure 7:**
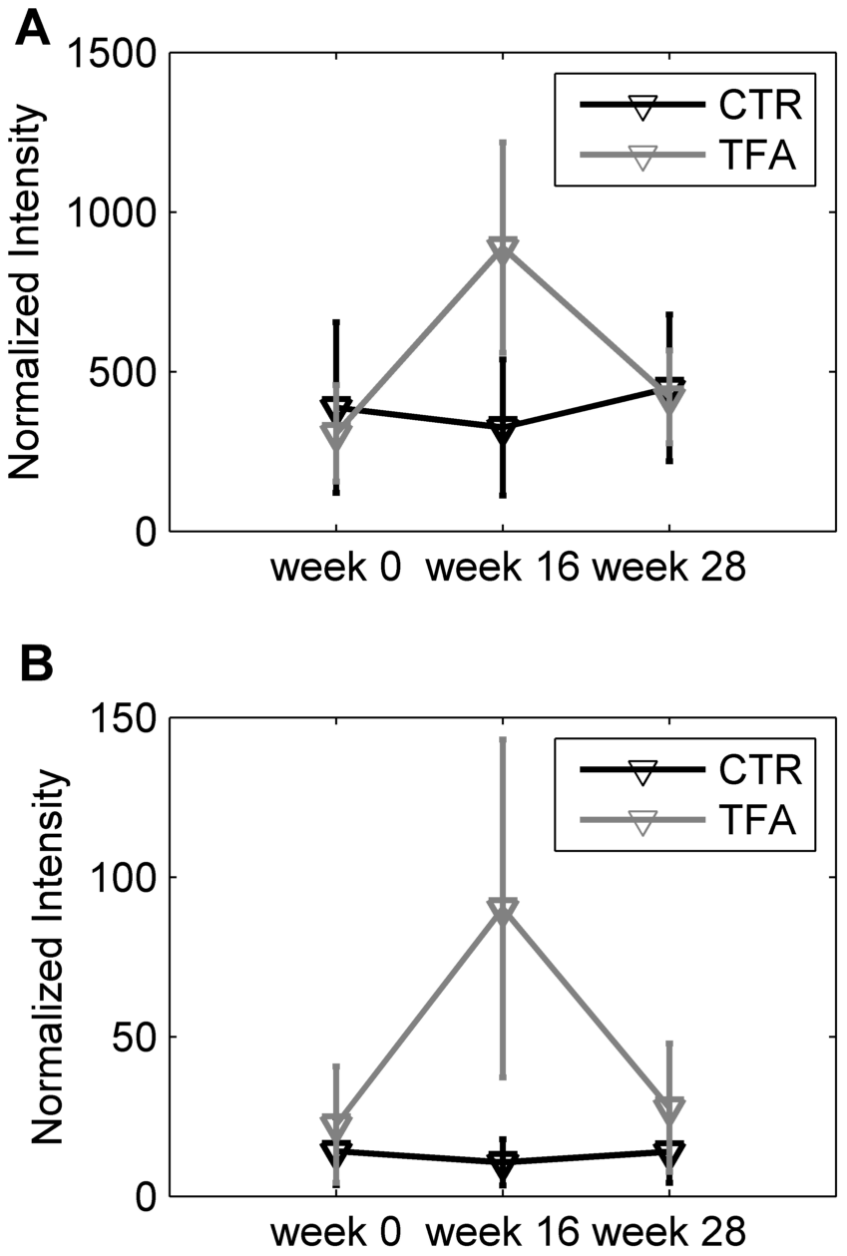
Normalized intensity for metabolites reflected by TFA intake. PC(40:7) (A) and SM(36:3) (B) at w0, w16 and w28. The values are the mean of samples in CTR and TFA groups. Each variable is normalized with the mean of the 9 recordings (at week 0, 16 and 28 with three OGTT time point recordings) for each subject.

In order to investigate whether the increase in specific lipids is temporary or remain for longer period, the measurements at w28 (i.e.12 weeks after the end of the intervention) were included. As mentioned earlier in this period all subjects had been under a weight loss program. The levels of SM(36:3) and PC(40:7) were increased at w16 and declined to the levels observed before intervention (w0) at w28 for the TFA group, whereas there was no change for the CTR group (Figure 7). The other markers in Table 1 exhibited similar trends (not shown). The standard deviation for the TFA group was higher at w16, which is related to varying individual responses to TFA intake despite the identical dose level for all participants in the TFA group.

Finally, to ascertain that the two major markers identified, SM(36:3) and PC(40:7), were genuine TFA markers in plasma we quantified them by a targeted lipidomics analysis of a subset of 12 samples from each group using appropriate internal standards. Under the lipidomics conditions used here the two markers emerged as significantly higher by factors of 16–40 in the period with *trans*-fat exposure and were very low before intervention or during control conditions after correction for internal standards. No other features emerged with similar strong contrasts and other PCs such as two PC(36:2) isomers did not differ between the two treatments. Some weaker markers of TFA exposures may possibly exist but that would need more extensive analysis of the full set to ascertain.

## Discussion

TFA has been banned in Denmark since 2004 and background levels of TFA in Danish citizens are therefore low, resulting only from residual exposures from ruminant fats [27]. This has made Denmark an ideal place for an intervention to investigate the short-term effects of TFA with a low background exposure. The level of exposure selected for the current study is high but not unattainable with high intakes of fast foods and snacks in countries where TFA in foods is not tightly regulated [28]. Several TFAs exist and in the current study, *trans*18:1 was almost exclusively present in the intervention oil [13]. From this well-controlled study of *trans vs. cis* C18:1 fat in overweight women we report that both ^1^H NMR and LC-MS plasma metabolic profiles were altered with TFA intake. In this as in many other studies consumption of TFA is related with an increased LDL to HDL ratio, which is considered as a powerful long-term predictor of cardiovascular disease [29]. Another outcome from NMR was elevated unsaturated lipid signals for the TFA group, which can be attributed to an increased level of unsaturated fatty acyl side chains in lipid species. The fatty acid composition of phospholipids in red blood cell membranes was reported by Bendsen et al. [13]. Their results did not reveal any significant alteration between the CTR and TFA groups with respect to the PUFA (or monounsaturated) fatty acid levels, except for a different content of TFA. Thus, this difference may be arising from unsaturation of other lipid groups such as triglycerides in the lipoproteins. Similarly, an elevated unsaturation in the NMR spectrum (δ 5.3–5.4 and δ 1.9–2.5) of HepG2 cell extracts exposed to TFA was mentioned by Najbjerg et al. [30] in which they concluded disturbed lipid storage efficiency with TFA intake.

The LC-MS profiles demonstrated elevated levels of a limited number of polyunsaturated long chain PCs (PC(40:7), PC(40:6), PC(38:4)) and of SM(36:3), which has the longest chain and the highest unsaturation among all detected SMs. Increased double bond formation was also supported by the NMR results. None of these markers were affected by the OGTT test, revealing that they are not necessarily only fasting state markers. TFA intake did not seem to have long term effects on the composition of plasma lipids, as their levels at w28 after intervention (after the weight loss period) were comparable to baseline (w0) levels as shown in Figure 7. We observed here SM(36:3) as a marker of TFA intake. This SM was present at very low levels in the non-TFA group indicating that it represents an unusual structure. An increased level of total plasma SMs has been associated with increased risk of atherosclerosis [31], [32] although the consequence in terms of cardiovascular risk has been debated [33]. In this study we observed an increase in only a single, minor SM having two C18 chains with one and two double bonds, respectively, either SM(d18:2/18:1) or SM(d18:1/18:2). The configuration (*cis* or *trans*) around the double bonds in these markers is unresolved and the atherogenic potential of this specific SM will need further investigation. There was no correlation between the concentration of this SM and any other SMs. We speculate that the marker observed here has a Δ9 or Δ 11 *trans*-fatty sphingosine chain containing a *cis*-double bond in the 3-position. This would result from Δ7 or Δ9- and other *trans*-hexadecanoic acids being a substrate for the slightly promiscuous serine palmitoyltransferase (EC 2.3.1.50) [34] to form a 3-ketodehydrosphingosine, which would then be reduced and acylated by oleyl-CoA followed by desaturation to form Cer(d18:2/18:1). This ceramide would act as a precursor to the SM(d18:2/18:1) formed by SM synthase (EC 2.7.8.27). We are not able to see the less polar products postulated here, not even by lipidomics, but the consequence of this hypothesis would be that after intakes of *trans*16:1 fatty acids it would be possible to observe the formation of a whole series of sphingolipids containing the unusual Δ9 or Δ11- *trans*- Δ3-*cis* C18:2 and other similar sphingosines with the *trans* double bond in other positions. In the current study *trans*16:1 was below the detection limit in the diet but it is likely that it is formed by β-oxidation of Δ9 or Δ 11-*trans*-18:1. In a study of 16:1 ruminant TFAs, the Δ9 was the dominating isomer but *trans* double bond isomers with the double bond at any carbon from position 3 up to 14 also existed [35]. The identity of our SM(d18:2/18:1) marker needs to be finally proven in separate studies, and if the assignment is correct the biological and especially neurological consequence of changing the usual *cis*-Δ3-sphingosines by an aberrant backbone must be elucidated.

We succeeded in identifying several PCs based on authentic standards and by a systematic pattern of RTs depending on chain length and saturation. Based on this pattern we could identify two PC's, PC(40:6) and PC(40:7), which were specifically increased in plasma following dietary TFAs, and PC(38:4), which tended to be increased as well. These PCs carry a C18:1 acyl side chain in one position and a long-chain PUFA chain in the other based on their CID fragmentation patterns. Since C20 and C22 acyl side chains in PCs are almost exclusively found in the sn-2 position in humans [36], it is most likely that the 18:1 is found in the sn-1 position. TFAs, including *trans*-vaccenic acid (Δ11-*trans*-18:1), sterically resemble saturated fatty acids and might therefore substitute for these in the sn-1 position. In agreement, the preferential incorporation of elaidic acid to the sn-1 chain of phospholipids has been reported in hepatocytes by Woldseth et al. [37]. In accordance, Wolf and Entressangles [38] showed that phospholipids from rat liver mitochondria modified *in vivo* had large quantities of elaidic acid esterified at the sn-1 position. We therefore propose that the species observed here are PC(*trans*18:1/22:5), PC(*trans*18:1/22:6) and PC(*trans*18:1/20:3). This hypothesis is supported by the previously reported elevated *trans*-18:1 residue levels in red blood cell phospholipids in the TFA group [13].

It is well known that TFA incorporate membrane phospholipids into plasma altering the packing of phospholipid and influencing the physical properties and responses of membrane receptors [39], [40]. TFA produce membrane properties more similar to those of saturated chains than those of acyl chains containing *cis* double bonds [40]. When incorporated into membrane phospholipids, TFA either replace existing saturated or *cis* unsaturated acyl chains. Harvey et al. [41] showed that both elaidic and linoelaidic acid integrated into phospholipids, mainly at the expense of myristic, palmitic, and stearic acids, without causing any net gain in total fatty acid levels. In our study, the published membrane phospholipid levels [13] revealed significantly decreased stearic acid (P = 0.04) and oleic acid (P = 0.02) abundance in the TFA group compared to CTR, suggesting replacement of those with elaidic acid. Although LC-MS based metabolomics did not show any decrease in PCs having one saturated fatty acyl chain, elaidic acid-containing specific PCs potentially increased in the TFA group. Many other researchers have investigated the variation of fatty acid composition in red blood cell PCs after TFA intake; however none of them reported the effect of TFA intake on specific PCs. Here, LC-MS based metabolomics demonstrated up-regulation of specific PCs with TFA. Moreover the inter-individual variation in the plasma level of these markers indicates that a variable response to the same dose of TFA may exist although any relation of our current markers to CVD risk needs confirmation in observational studies.

The TFA markers, PC(*trans*18:1/20:3), PC(*trans*18:1/22:4) and PC(*trans*18:1/22:5), preferentially integrated into PCs all contain PUFA in the sn-2 position. There was no difference in the dietary intake of PUFAs in the two diet groups [13], so the preferred presence of these specific acyl chains together with *trans*18:1 would need an explanation. The two minor markers have peaks with a RT slightly different from the main, 18:0 containing PC(40:6) and PC(38:4) peaks (Figure 1), indicating that they may be detectable due to better signal-to-noise ratio for these specific compounds, but the more prominent PC(40:7) marker is actually dominating the only PC(40:7) peak observed and the level in the non-TFA group is quite low. This is not surprising since this compound in general would be a minor PC because it violates the general rule of saturated sn-1 and unsaturated sn-2 acyl chains and because no C22 fatty acid with seven double bonds exists in human lipids. Other minor TFA-containing PCs may therefore exist but with RTs that fall on top of major PCs so that they are not detected as markers. However, it is still noticeable that PC(40:7) is so abundant. It forms a large peak comparable to other major PCs, indicating a facilitated formation. We also found evidence for the presence of the even longer PC(44:9). These observations could either indicate that there is a general up-regulation in the formation of long-chain PUFAs after TFA intake and/or that these fats are specifically mobilised into PC as a result of TFA exposure. It has been shown that the acyl chain distribution is very similar in plasma and erythrocyte membranes, indicating that plasma PCs may be a surrogate marker for membrane composition. Indeed, most plasma PCs may be abstracted from the membranes in contact with blood. Increased formation of long-chain PUFAs has been observed in adipose tissue membranes in overweight individuals [42], resulting from increased elongase and desaturase activities. This phenomenon is likely due to compensation for the increased fat load in the adipocytes in order for them to remain functional, despite their enlargement during weight gain [42]. TFA resembling saturated fatty acids may therefore negatively affect adipose tissue function leading to a response similar to that seen during weight gain with increased formation of long-chain PUFA's. This is supported also by an increased unsaturation in the NMR spectra for the TFA group, yet the FA composition of red blood cell phospholipids did not show any overall significant increase in PUFA [13]. Further investigation of the PUFA distribution among specific membrane PCs is therefore needed in order to confirm this hypothesis.

As previously mentioned, phospholipids containing TFA behave similar to saturated fatty acids rather than to their *cis* monounsaturated isomers. It has been reported that *trans*-acyl chains adopt extended configurations similar to saturated acyl chains, allowing better interaction with the cholesterol molecule compared with their *cis* analogs [43]. These effects could be contributing factors in modulating cholesterol homeostasis, and as such, may be part of the explanation of the elevation of LDL cholesterol by a TFA-rich diet [43] which was demonstrated by NMR. Although TFA has properties similar to those of saturated fatty acids and also substitute for saturated fatty acids in membrane lipids, it has been confirmed in a meta-analysis that TFA raises levels of LDL more than an equal amount of saturated fatty acids. This demonstrates the effect on LDL levels is much larger when TFAs are compared with their *cis* analogs [6].

### Conclusions

This study was established to investigate the effect of 18:1 TFA intake on plasma metabolites using an untargeted approach. As results demonstrate specific lipid molecular species in plasma were formed as a result of TFA exposure and all belong to the SM and PC polar lipids that exist in plasma in equilibrium with the plasma membranes. From those, SM(d18:2/18:1) and PC(*trans*18:1/22:6) may be a general plasma marker of exposure to TFAs. We observed a variable individual marker response to the same TFA dose and the consequence of this response variation should be tested in further studies. We could also confirm that TFA exposure leads to increased plasma LDL. Further studies with other specific exposures to 16:1 and 18:2 TFAs would give further insight into the general and specific lipid markers of TFA exposure.

## Supporting Information

Figure S1 **CONSORT flow diagram**
(PDF)

Protocol S1 Clinical trial protocol
(PDF)
