# Supplementary figures and images for: Effect of trans Fatty Acid Intake on LC-MS and NMR Plasma Profiles
Source: PLoS One. 2013 Jul 29;8(7):e69589. doi: 10.1371/journal.pone.0069589 (PMC3726671; doi:10.1371/journal.pone.0069589)

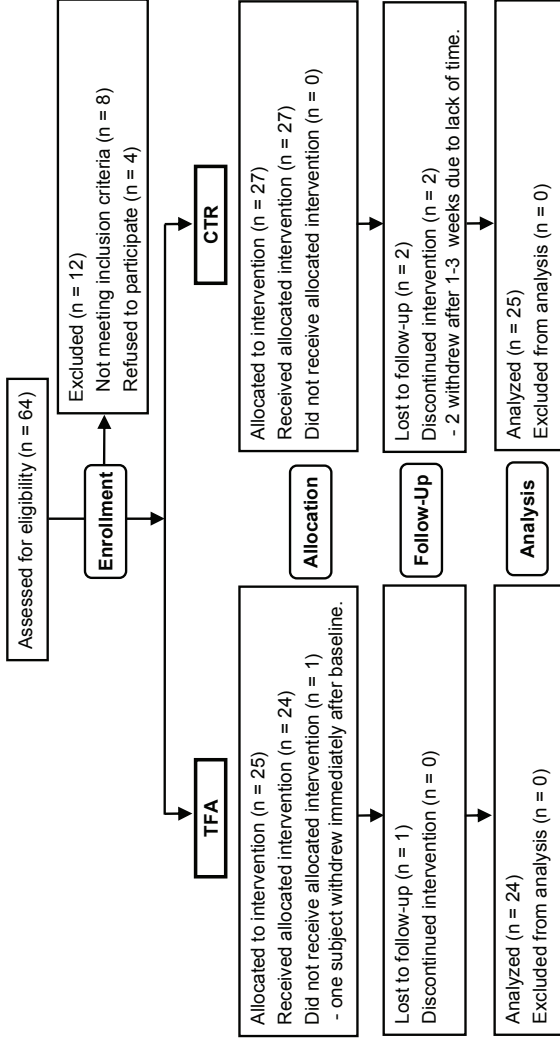

Supplement: Figure S1 — CONSORT flow diagram (PDF) [file pone.0069589.s001.pdf]
