# Supplementary material for: Effect of trans Fatty Acid Intake on LC-MS and NMR Plasma Profiles
Source: PLoS One. 2013 Jul 29;8(7):e69589. doi: 10.1371/journal.pone.0069589 (PMC3726671; doi:10.1371/journal.pone.0069589)
Supplement: Protocol S1 — Clinical trial protocol (PDF) [file pone.0069589.s002.pdf]

Protokol:

COBRA

Copenhagen Obesity Risk Assessment study

Et dobbeltblindet, randomiseret kostinterventionsforsøg, der undersøger effekten af et højt indtag af transfedtsyrer på abdominal fedme samt risikomarkører for udvikling af type 2 diabetes, og hjertekarsygdom.

Index

1. Resumé **4**
2. Flow diagram **5**
3. Indledning **6**
4. Design **8**
4.1. Studiedesign 8
5. Forsøgspersoner **8**
5.1. Antal forsøgspersoner 8
5.2. Inklusionskriterier, hovedstudie 9
5.3. Inklusionskriterier, normalvægtige referencer 9
5.4. Eksklusionkriterier, hovedstudie og normalvægtige referencer 9
5.5. Kriterier for udelukkelse i studieforløbet 9
6. Studieforløb **10**
7. Metoder og undersøgelser **10**
7.1. Planlagte besøg 10
7.2. Stratificering 14
7.3. Metoder, primære effektparametre 14
7.4. Metoder, sekundære effektparametre 15
7.5. Metoder, sikkerhedsparametre 16
7.6. Metoder, kontrol og bestemmelse af compliance 17
7.7. Håndtering af biologisk materiale 17
8. Interventionskost **17**
8.1. Industrielt produceret transfedtsyreberiget kost 18
8.2. Kontrolkost 18
8.3. Opbevaring af forsøgsmateriale 18
9. Registrering af sygdom og medicinindtag **18**
9.1. Baseline 18
9.2. Forsøgsforløb 18
10. Bivirkninger **18**
10.1. Definitioner 18
10.2. Rapportering af bivirkninger 18
11. Case Report Forms (CRF) **19**
11.1. Regler for brug af CRF 19
11.2. Rettelser i CRF 19

12. Databehandling **19**
12.1. Kvalitetssikring af data 19
12.2. Statistiske metoder 19
13. Etik **20**
13.1. Videnskabsetisk komité bedømmelse 20
13.2. Etisk gennemførelse af studiet 21
13.3. Mulige fordele af studiet og sikkerhedsaspekter 21
13.4. Samtykkeerklæring 22
13.5. Forsøgspersoners privatliv 22
14. Hvervning af deltagere **22**
15. Tilgængelighed af oplysninger for forsøgspersoner **22**
16. Offentliggørelse af forsøgsresultater **22**
17. Økonomiske forhold **22**
18. Partnere **23**
19. Referencer **24**

Appendiks 1: Lægmandsbeskrivelse **26**
Appendiks 2a: Deltagerinformation **28**
Appendiks 2b. Deltagerinformation, normalvægtige referencer **34**
Appendiks 3: ”Dine rettigheder som forsøgsperson i et biomedicinsk forskningsprojekt” **39**
Appendiks 4: Procedure for afgivelse af mundtlig deltagerinformation **40**
Appendiks 5: Samtykkeerklæring **41**
Appendiks 6a: Annoncetekst til hvervning af deltagere **43**
Appendiks 6b: Annoncetekst til hvervning af normalvægtige referencer **44**
Appendiks 7: Skema for registrering af fysisk aktivitet **45**

1. Resumé

Formål

Hos moderat overvægtige postmenopausale kvinder at undersøge om industrielt producerede transfedtsyrer (IP-TFA) i en daglig dosering på 15 g i 16 uger fører til påvirkning af abdominal fedme, samt risikomarkører for type 2 diabetes og hjertekarsygdom, der kan belyse den overdødelighed, som er beskrevet i prospektive epidemiologiske studier hos personer med et dagligt indtag af få gram transfedtsyrer, gennem en årrække.

Primært mål

- At bestemme ændring i: Fedtaflejring i leveren, insulinvirkning (beregnet som Insulin Sensitivity Index Composite (ISI(composite)) under oral glukose tolerans test), samt lipidæmi (beregnet som LDL-kolesterol/HDL-kolesterol-ratio).

Associerede mål for dyslipidæmi er ændring i totalkolesterol (TC), TC/HDL-kolesterol-ratio, HDL-kolesterol, LDL-kolesterol samt triglycerid. Associerede mål for insulinresistens er ISI Homeostatic Model Assessment (HOMA), faste glukose, faste insulin og glukosetolerans.

Sekundære mål

- At belyse effekten af IP-TFA på abdominal fedme, samt andre risikomarkører for type 2 diabetes og hjertekarsygdom: kropsfedtfordeling, visceralt fedt, intramuskulært fedt, insulinfølsomhed, inflammatoriske markører i blod samt fedtvæv, ceramid i fedtvæv og hjerterytmevariabilitet.
- At foretage metabolisk profilering på blod, urin og fedtvæv ed formålet at indentificere mulige biomarkører for hhv. indtag af og metaboliske effekter af IP-TFA.

Studiedesign

Enkeltcenter randomiseret, dobbeltblindet, kontrolleret, parallelgrupperet design, hvor ca. 48 abdominalt fede, moderat overvægtige postmenopausale kvinder i alderen 45-70 år randomiseres til én interventionsgruppe og én kontrolgruppe (24 personer/gruppe). I interventionsgruppen beriges den daglige kost med 15 g IP-TFA. Fedtsyreberigelsen substituerer anden fedt i kosten. Til sammenligning anvendes en kontrolgruppe, der indtager en kost, hvor transfedt er erstattet af *cis*-monoumættet fedt.

Kostinterventionsperioden er på 16 uger. Ved kostinterventionsperiodens ophør deltager forsøgspersonerne i et 12 ugers intensivt vægttabsprogram under diætistvejledning. Herefter afsluttes med et opfølgningsbesøg efter i alt 28 uger.

Studiedeltagere: Cirka 60 abdominalt fede kvinder indkaldes til screening og de randomiseres til interventions- og kontrolgruppen. Det forventes, at ca. 52 personer gennemfører screening.

Der rekrutteres endvidere 20 personer, som skal repræsentere en normalvægtig referencegruppe, der kun gennemgår baselineundersøgelser.

Compliance

Vurderes ved inkorporering af fedtsyrer i form af fosfolipider (PL) i cellemembranen i de røde blodlegemer (RBC) og som triglycerid i fedtbiopsier udtaget fra abdominalt fedtvæv.

Sikkerhed

Bivirkninger, blodtryk, hæmatologi og biokemiske analyser.

Kost

Forsøget udføres som en kostintervention, hvor personerne i de pågældende grupper, som erstatning for anden kost, får udleveret særligt fremstillet mad, der indeholder de ønskede mængder af fedtsyrer.

2. Flow diagram

	Screening	Baseline	Kostintervention			Interv. slut	Vægttabsprogram					Opfølgning
**Uge nr.**	**-12 - -4**	**0**	**4**	**8**	**12**	**16**	**18**	**20**	**22**	**24**	**26**	**28**
**Besøg nr.**	**1**	**2**	**3**	**4**	**5**	**6**	**7**	**8**	**9**	**10**	**11**	**12**
Informeret samtykke	x											
In- og eksklusionskriterier	x											
Demografi	x											
Sygehistorie	x											
Højde	x											
Vægt	x	x	x	x	x	x	x	x	x	x	x	x
Talje/hofte	x	x		x		x						x
Blodtryk	x	x		x		x						x
Tidligere/samtidig medicin	x	x	x	x	x	x	x	x	x	x	x	x
Bivirkninger		x	x	x	x	x	x	x	x	x	x	x
Randomisering		x										
Blodprøver (fastende)	x	x		x		x						x
- *Hæmatologi*	x					x						x
- *Biokemi (lever-markører)*	x					x						x
- *Lipider (TG, HDL-C, LDL-C, Total-C)*	TG + TC	x		x		x						x
- *Glukose, insulin og C-peptid*	Glukose			x								
- *RBC (membran PL fedtsyreprofil)*		x		x		x						x
- *hCRP*		x		x		x						x
- *Adiponektin*		x		x		x						x
- *Cytokiner (TNF-alpha, IL-6, IL-8)*		x		x		x						x
3 t OGTT m glukose, insulin, C-peptid og FFA		x				x						x
Inkretiner og væksthormon under OGTT		x				x						x
Urinopsamling (24 timer m. PABA-kontrol)		x				x						x
Fedtbiopsi, abdominal (m. lokal-anæstesi)		x				x						x
DEXA (kropssammensætning) & sagittalhøjde		x				x						x
24 timer Holter-monitorering (HRV)		x				x						x
MR-scanning (lever-, intramusk. og visceralt fedt)		x				x						x
Udlevering af mad (én gang/uge)		xxxx	xxxx	xxxx	xxxx							
3-dages vejet kostregistrering	x					x						x
3-dages registrering af fysisk aktivitet	x					x						x
Kostinstruktion - vægttabsprogram							x	x	x	x	x	x
Compliance tjek (dagbog)			x	x	x	x	x	x	x	x	x	x

3. Indledning

En række undersøgelser indenfor de senere år har vist, at et højt indtag af industrielt producerede transfedtsyrer øger risikoen for udvikling af hjertekarsygdom. I en metaanalyse af 4 kohortestudier fra USA, Finland, og Holland blev det således observeret, at en stigning i indtaget af transfedtsyrer på 2 E % giver en risikoforøgelse for kardiovaskulære episoder på ca. 25 % (1). Den prospektivt observerede effekt i Nurses' Health study med 14 års opfølgning af mere end 80.000 kvinder var endnu større, med en risikoforøgelse på 93% (2). Observationelle undersøgelser har peget på, at indtag af transfedtsyrer også er relateret til øget forekomst af type 2 diabetes hos kvinder. I Nurses' Health Study var 2 E% transfedtsyrer således ledsaget af en næsten 40% øget incidens af type 2 diabetes (3).

Der er flere potentielle mekanismer, hvorved transfedtsyrer kan accelerere udviklingen af hjertekarsygdom. Det er gentagne gange i både observationelle og randomiserede studier vist, at transfedtsyrer øger ratioen mellem LDL- og HDL-kolesterol i blodet, hvilken er en vigtig risikomarkør for hjertekarsygdom (4;5). Effekten af transfedtsyrer herpå kan imidlertid blot forklare en ca. 5 % risikoforøgelse (6), og der synes således at være andre uheldige virkninger af transfedtsyrer end påvirkning af blodets lipid- og lipoproteinforhold.

I modsætning til transfedtsyrer er n-3 flerumættede fedtsyrer (fra fiskeolie) fundet at have en gavnlig effekt på blodets lipider, samt på hjerterytmevariabilitet (HRV)(7;8). Vi har derfor opstillet den hypotese, at transfedtsyrer i kosten på cellulært niveau blandt andet ved at konkurrere med n-3 fedtsyrer er en selvstændig risikofaktor for ustabil hjerterytme og dermed for pludselig hjertedød. En påvisning af en sådan effekt vil give et væsentligt bidrag til at forklare de ovenfor anførte forskelle mellem observeret risikoforøgelse for hjertekarsygdom og den risiko, som påvirkning af blodets lipider udgør.

Tidligere har vi fundet ingen eller ringe effekt af hhv. transfedtsyrer og n-3 flerumættede fedtsyrer på hjerterytmevariabilitet og blodlipider i et 8-ugers randomiseret kontrolleret kostinterventionsstudie (9). Den relativt korte interventionsperiode, samt den omstændighed at studiepopulationen var raske, moderat fysisk aktive, ikke-abdominalt fede og normalvægtige mænd, kan muligvis forklare, at der ikke blev fundet en effekt af transfedtsyrer. Det er uvist, hvor lang tid der går, før transfedtsyreindtaget evt. kan påvirke HRV og det er derfor muligt, at 8 uger er for kort tid til at observere effekter på HRV hos raske. Derfor vil vi nu gennemføre et 16-ugers kostinterventionsstudie med præobese kvinder. Vi ønsker udelukkende at inkludere kvinder, idet den foreliggende evidens fra observationelle studier indikerer, at kvinder muligvis er mere påvirkelige af et højt indtag af transfedtsyrer end mænd (1;3).

I dette studie ønsker vi endvidere at inkludere en række øvrige målinger, idet nyere forskning tyder på, at en del af den skadelige effekt af transfedtsyrer muligvis kan udspille sig gennem øget abdominal fedme, insulinresistens samt andre end de traditionelle risikofaktorer for hjertekarsygdom, og sammenhængen støttes af et flere-årigt interventionsstudie på aber (10;11). Sidstnævnte kontrollerede kostinterventionsstudie viste, at et højt indtag af transfedtsyrer øger aflejring af intra-abdominalt fedt sammenlignet med et iso-kalorisk indtag af tilsvarende cis-umættede fedtsyrer. Indtag af transfedtsyrer inducerede endvidere vægtøgning og var associeret til nedsat insulinfølsomhed (10). At indtag af transfedtsyrer kan føre til øget abdominal fedme tyder resultaterne i det prospektive Health Professionals' Follow-up Study også på, idet selvrapporteret taljeomkreds i multivariatanalyse var positivt associeret til transfedtindtag efter 9 års opfølgning, selv når der korrigeredes for BMI (11). Idet det er tvivlsomt, hvorvidt ændringer i abdominalt fedt kan påvises efter blot 16 ugers kostintervention, vil vi måle fedtaflejring i leveren, som der er visse holdepunkter for kan være en mere sensitiv indikator for en begyndende sundhedsskadelig fedtaflejring end abdominalt fedt (12). Endvidere er fedtvævsaflejring i f.eks. lever og muskel som følge af øget transfedtsyreindhold i kosten en mulig mekanisme bag insulinresistens (13).

Der er en tæt forbindelse mellem insulinresistens og intra-abdominal fedme, subklinisk systemisk inflammation, dyslipidæmi og atherosclerose (14;15). Hos normalvægtige, raske forsøgspersoner har randomiserede undersøgelser ikke vist effekt af transfedtsyrer på insulinfølsomheden (16;17). Disse studier er dog af kortere varighed (4 uger). Det er uvist, hvor lang tid der går, før transfedtsyreindtaget afspejler sig i

strukturelle lipider, som er bestemmende for insulinfølsomhed (f.eks. membran fosfolipider i skeletmuskulaturen), og det er derfor muligt, at 4 uger er for kort tid til at observere effekter på insulinfølsomheden hos raske. Der findes nogen evidens for, at transfedtsyrer sænker det postprandielle insulinrespons hos overvægtige, og hos patienter med type 2 diabetes (18;19). I nærværende 16-ugers kostinterventionsstudie med præobese forsøgspersoner er derfor inkluderet peroral glukosebelastning (OGTT) med måling af plasma-insulin, -glukose og -C-peptid, som en markør for ændring i insulinfølsomhed.

Nyere studier peger endvidere på, at transfedtsyrer har proinflammatoriske effekter (20-22) og niveauet af transfedtsyrer i cellemenbraner i røde blodlegemer er fundet at være associeret med systemisk inflammation (23). Ceramid i fedtvæv er associeret til cytokinekspression (24), derfor bestemmes udover cytokinekspression endvidere triglycerid- og ceramidindhold og -komposition i fedtvæv.

Formålet med nærværende studie er i et kontrolleret design at måle effekten af daglig indtagelse af IP-TFA på udvikling af abdominal fedme samt risikomarkører for type 2 diabetes, og hjertekarsygdom, sammenlignet med en kontrolkost, hvor transfedt er erstattet af *cis*-monoumættet fedt.

Primære formål er, at undersøge i hvor høj grad en kost med et højt indhold af IP-TFA sammenlignet med en IP-TFA fri kontrolkost påvirker:

- Blodets lipider, særligt ratioen mellem LDL- og HDL-kolesterol
- Insulinsekretion, insulinfølsomhed og glukosetolerans
- Aflejring af fedt i leveren

Sekundære formål er, at undersøge hvordan de to kosttyper påvirker:

- Kropsvægt og kropsfedtfordeling
- Cytokin-ekspression i fedtvæv
- Ceramid i fedtvæv
- Hjerterytmevariabilitet

En eventuel effekt af IP-TFA på disse risikomarkører kan muligvis udspille sig gennem inkorporering af fedtsyrerne i fedtvæv. Derfor vil inkorporering af transfedtsyrer i fedtvævet også blive undersøgt.

Desuden er det ønsket at foretage metabolisk profilering på blod, urin og fedtvæv.

Med hensigten at kunne etablere et mål for hvor "dysregulerede" de overvægtige forsøgsdeltagere er ved baseline og kunne relatere de ændringer der måtte ske under kostinterventionen i forhold til en normal referencegruppe, inkluderes en referencegruppe af matchede normalvægtige kvinder, som kun undersøges én gang og ikke gennemgår kostintervention. Disse referencedata vil desuden godtgøre, i hvilken grad opfølgningsperioden med diætistkontrolleret vægttabsprogram er i stand til at "normalisere" de overvægtige forsøgsdeltagere på endepunkterne.

Med dette studie ønskes det afklaret i et kontrolleret design, hvorvidt sundhedsskadelige effekter af IP-TFA kan dokumenteres. Såfremt TFA har skadelige effekter, vil studiet desuden kunne afdække, hvorledes disse opstår. Informationer om effekten af indtag af transfedtsyrer fra dette studie, kan anvendes ved fremtidige officielle anbefalinger og af politikere til regulering af IP-TFA-tilgængeligheden i fødevarer. En sådan regulering vil potentielt kunne forebygge udvikling af metabolisk syndrom, type 2 diabetes og hjertekarsygdom i befolkningen.

4. Design

4.1. Studiedesign

Studiet er et dobbeltblindet randomiseret kostinterventionsstudie, hvor raske, men abdominalt fede og moderat overvægtige (BMI 25-32) postmenopausale kvinder i alderen 45-70 år i 16 uger udsættes for en kost beriget med IP-TFA. Før, under og efter kostberigelsen måles abdominal fedme, samt risikomarkører for udvikling af type 2 diabetes, og hjertekarsygdom. Interventionsgruppen sammenlignes med en kontrolgruppe, der får en kost, hvor transfedtsyrer er erstattet af *cis*-monoumættet fedt, primært oliesyre. Studiet vil således have to arme, og forsøgspersonerne vil blive ligeligt randomiseret til hver arm i studiet.

Interventionskosten vil blive tilberedt med fedt, der erstatter andet kostfedt i forsøgspersonernes habituelle kost. I den transfedtsyreberigede kost vil den daglige kost blive beriget med 15 g transfedtsyrer, som vil være en blanding af *trans*18:1 og 18:2 isomerer.

Studiet består af et screeningsbesøg, hvor forsøgspersonernes egnethed til at indgå i studiet vurderes. Efter randomisering indtager deltagerne én af de 2 kosttyper i en 16 ugers periode. Dernæst gennemgår forsøgspersonerne et 12 ugers vægttabsprogram under vejledning af professionelle diætister. Ved vægttabsprogrammets afslutning, 12 uger efter kostinterventionen, er der planlagt en efterundersøgelse (besøg 12) for at registrere, hvor hurtigt forsøgsdeltagerne vender tilbage til deres vanlige tilstand (se diagram nedenfor).

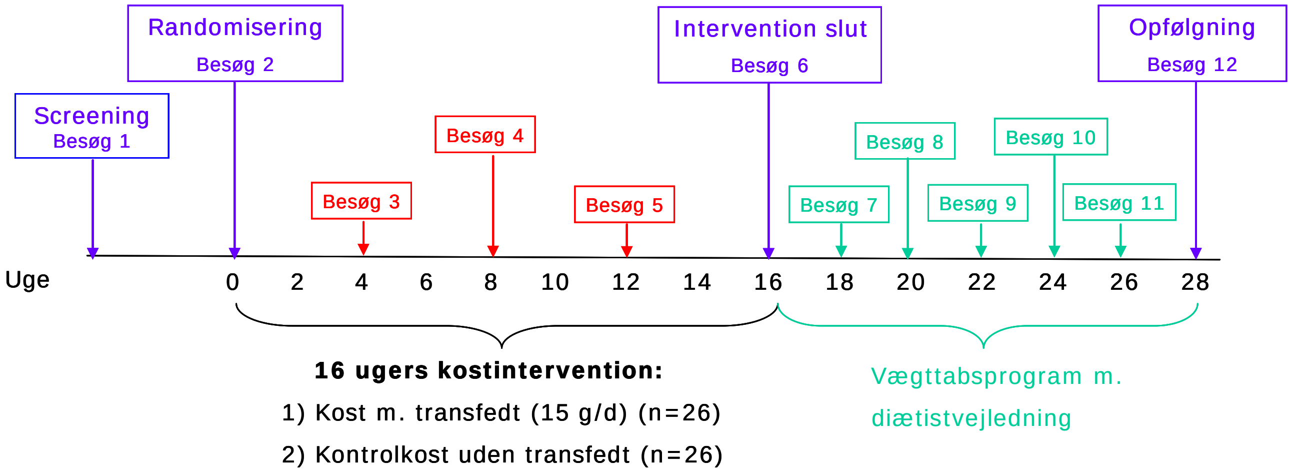

Der er således i alt 12 besøg, heraf 6 besøg i den kontrollerede del af studiet: et screeningsbesøg et baselinebesøg 1-12 uger herefter, og besøg efter henholdsvis 4, 8, 12 og 16 ugers kostintervention. Dernæst følger 5 diætistkonsultationer i det efterfølgende ukontrollerede vægttabsprogram. Der afsluttes med et opfølgningsbesøg efter i alt 28 uger.

Endvidere vil 20 raske og normalvægtige forsøgspersoner blive indkaldt til screening og baseline-undersøgelser. Disse personer vil tjene som referencegruppe.

5. Forsøgspersoner

5.1. Antal forsøgspersoner

Det er ønsket at 24 kvinder i hver af de 2 arme, i alt 48 forsøgspersoner, gennemfører studiet. Dette forventes at kræve screening af ca. 60 forsøgspersoner og en randomisering af ca. 52 personer (26 i hver arm).

Desuden vil 20 normalvægtige kvinder, såkaldte referencedeltagere, gennemføre baselinebesøget.

5.2. Inklusionskriterier, hovedstudie

- Kvinder
- Alder: 45-70 år
- BMI: 25-32 kg/m^2
- Taljeomkreds: ≥ 80 cm (Hvilket er den International Diabetes Federation´s 2005 kriterier for abdominal fedme / definition af metabolisk syndrom)
- Har ikke haft menstruation i minimum 1 år.

5.3. Inklusionskriterier, normalvægtige referencer

- Kvinder
- Alder: 45-70 år
- BMI: 19-23 kg/m^2
- Taljeomkreds: < 80 cm
- Har ikke haft menstruation i minimum 1 år.

5.4. Eksklusionkriterier, hovedstudie og normalvægtige referencer

- Diabetes mellitus eller anden alvorlig kronisk sygdom
- Tidligere eller nuværende betydelig kardiovaskulær sygdom
- Hypertension (siddende diastolisk blodtryk > 100 eller systolisk blodtryk > 160 mmHg) [Tjekkes ved screening besøg]
- Hyperglykæmi (fastende plasma glukose > 7.0 mmol/l) [Tjekkes ved screening besøg]
- Hypertriglyceridæmi (fastende TG > 3 mmol/l) [Tjekkes ved screening besøg]
- Hyperkolesterolæmi (fastende TC > 7 mmol/l) [Tjekkes ved screening besøg]
- Abnorme fund i hæmatologi og/eller biokemi, som af lægelige grunde ekskluderer [Tjekkes ved screening besøg]
- Kendt eller mistænkt misbrug eller afhængighed af medicin, alkohol eller euforiserende stoffer
- Vægtændring på > 3 kg inden for 2 måneder før screening besøg
- Brug af anti-hypertensionsmedicin, som påvirker blodlipidmetabolismen
- Brug af fiskeolie som kosttilskud senere end en måned før studiets start
- Deltagelse i kostinterventionsforsøg inden for 3 måneder før studiets start
- Fødevareintolerance eller fødevareallergi, som vurderes at være uforeneligt med deltagelse i forsøget
- Tobaksrygning inden for de seneste 6 måneder før studiets start.
- Moderat fysisk aktivitet > 4 timer om ugen
- Bloddonation indenfor de sidste 3 måneder før påbegyndelse af forsøget. Bloddonation under forsøget tillades ikke.

5.5. Kriterier for udelukkelse i studieforløbet

- Efter eget ønske: Alle forsøgspersoner har ret til at trække sig ud af forsøget på ethvert givet tidspunkt og af enhver given grund.

- Compliance: En forsøgsperson kan udelukkes fra forsøget, hvis projektlederen vurderer, at forsøgsdeltageren åbenlyst ikke følger studiets retningslinier.
- Bivirkninger: Der forventes ikke at fremkomme bivirkninger foranlediget af deltagelse i forsøget.

6. Studieforløb

Planlagt rekrutteringsperiode: Primo 2008

Planlagt inklusion af første forsøgsperson: 2. kvartal 2008

Planlagt afslutning af sidste forsøgsperson: 2. kvartal 2009

Det samlede studie forventes at kunne afvikles på 14 måneder

7. Metoder og undersøgelser

7.1. Planlagte besøg

Der vil være i alt 12 besøg (fremmøde på undersøgelses-centeret). Syv af disse besøg vil være regulære besøg, hvor der vil blive foretaget en række kliniske undersøgelser: Screening (besøg 1), baseline (besøg 2), besøg efter 4, 8, 12 og 16 ugers intervention, samt et opfølgende besøg efter 12 uger. Fem besøg vil alene bestå af diætistkonsultation, som vil ligge 2, 4, 6, 8 og 10 uger efter kostinterventionsperiodens afslutning.

Besøgene bør finde sted inden for følgende afvigelse fra den planlagte tidsplan:

Besøg 2: 1-12 uger efter besøg 1
Besøg 3: 4 uger ± 5 dage efter besøg 2
Besøg 4: 8 uger ± 5 dage efter besøg 2
Besøg 5: 12 uger ± 5 dage efter besøg 2
Besøg 6: 16 uger ± 5 dage efter besøg 2
Besøg 7: 2 uger ± 3 dage efter besøg 6
Besøg 8: 4 uger ± 3 dage efter besøg 6
Besøg 9: 6 uger ± 3 dage efter besøg 6
Besøg 10: 8 uger ± 3 dage efter besøg 6
Besøg 11: 10 uger ± 3 dage efter besøg 6
Besøg 12: 12 uger ± 7 dage efter besøg 6

Ved besøg 1, 2, 3, 4, 5, 6 og 12 anmodes forsøgspersonerne om at møde fastende, efter kun at have drukket en halv liter vand siden midnat til besøget næste morgen.

Ved et informationsmøde inden besøg 1 (screening) vil forsøgspersonerne blive informeret om forsøgets formål.

7.1.1. Besøg 1: Screening

Forsøgspersonerne vil blive tildelt et screeningsnummer og følgende vil blive foretaget:

- Indhentning af informeret samtykke (mundtligt og skriftligt samtykke), hvis ikke allerede indhentet ved informationsmøde.
- Tjek af in- og eksklusionskriterier.
- Demografiske data: fødselsdata
- Sygehistorie
- Oplysning om eventuelt medicinindtag
- Måling af højde og kropsvægt
- Måling af talje- og hofteomkreds
- Måling af blodtryk efter 10 minutters hvile i siddende stilling

- Blodprøvetagning til hæmatologi
- Blodprøvetagning til biokemiske undersøgelser
- Blodprøvetagning til glukose, total kolesterol og triglycerider
- Instruktion i 3-dages registrering af fysisk aktivitet (vil evt. foregå ved separat besøg)
- Instruktion i 3-dages vejet kostregistrering (vil evt. foregå ved separat besøg)
- Kostinstruktion (mundtlig og skriftlig) for interventionsperioden (instruktioner i anvendelse af den udleverede kost) (vil evt. foregå ved separat besøg)
- Udlevering af urin-dunke til døgn-opsamling og PABA-tabletter (vil evt. foregå ved separat besøg)

7.1.2. Besøg 2: Baseline

Besøg 2 skal finde sted 1-12 uger efter besøg 1. Besøg 2 vil strække sig over 2 dage.

Dette besøg påbegynder den dobbeltblindede randomiserede kostinterventionsperiode.

Følgende vil blive foretaget dag 1:

- Påbegyndelse af 24 timers urinopsamling og indtag af PABA-tabletter
- Påsætning af HOLTER-apparat til måling af Hjerte Rytme Variabilitet (HRV)

Følgende vil blive foretaget dag 2:

- Registrering af ændringer i medicinforbrug
- Registrering af uønskede hændelser
- Måling af kropsvægt
- Måling af talje- og hofteomkreds
- Blodprøvetagning til hæmatologi
- Blodprøvetagning til biokemi
- Blodprøvetagning til lipider
- Blodprøvetagning til fedtsyresammensætning af membran fosfolipider i RBC
- Blodprøvetagning til adipokiner og cytokiner
- Blodprøvetagning til metabolisk profilering
- Måling af blodtryk efter 10 minutters hvile i siddende stilling
- Aflevering af 24 timers urinopsamling
- Fedtvævsbiopsi
- Helkrops DEXA-skanning + sagittal-højde (måling af kropssammensætning og abdominal fedme)
- OGTT med glukose-, C-peptid- og insulinmåling mv. fastende og over 3 timer. Spoturin udtages før og efter testen til metabolisk profilering.
- Afmontering af HOLTER-apparat til måling af Hjerte Rytme Variabilitet (HRV)
- MR skanning
- Udlevering af interventionskost

7.1.3. Besøg 3

Besøg 3 skal finde sted 4 uger ± 5 dage efter besøg 2.

Følgende vil blive foretaget:

- Registrering af medicinforbrug
- Registrering af uønskede hændelser / bivirkninger
- Måling af kropsvægt
- Tjek af kostdagbog/compliance
- Udlevering af interventionskost

7.1.4. Besøg 4

Besøg 4 skal finde sted 8 uger ± 5 dage efter besøg 2.

Følgende vil blive foretaget:

- Registrering af medicinforbrug
- Registrering af uønskede hændelser / bivirkninger
- Måling af kropsvægt
- Måling af talje- og hofteomkreds
- Måling af blodtryk efter 10 minutters hvile i siddende stilling
- Blodprøvetagning til lipider
- Blodprøvetagning til fedtsyresammensætning af membran fosfolipider i RBC
- Blodprøvetagning til adipokiner og cytokiner
- Blodprøvetagning til glukose, insulin og c-peptid
- Tjek af kostdagbog/compliance
- Udlevering af interventionskost

7.1.5. Besøg 5

Besøg 5 skal finde sted 12 uger ± 5 dage efter besøg 2.

Følgende vil blive foretaget:

- Registrering af medicinforbrug
- Registrering af uønskede hændelser / bivirkninger
- Måling af kropsvægt
- Tjek af kostdagbog/compliance
- Udlevering af interventionskost
- Instruktion i 3-dages registrering af fysisk aktivitet i dagene op til besøg 6
- Instruktion i 3-dages vejet kostregistrering i dagene op til besøg 6

7.1.6. Besøg 6: Afslutning af kostintervention

Besøg 6 skal finde sted 16 uger ± 5 dage efter besøg 2. Besøg 6 vil strække sig over 2 dage:

Følgende vil blive foretaget dag 1:

- Påbegyndelse af 24 timers urinopsamling
- Påsætning af HOLTER-apparat til måling af Hjerte Rytme Variabilitet (HRV)

Følgende vil blive foretaget dag 2:

- Registrering af medicinforbrug
- Registrering af uønskede hændelser
- Måling af kropsvægt
- Måling af talje- og hofteomkreds
- Blodprøvetagning til hæmatologi
- Blodprøvetagning til biokemi
- Blodprøvetagning til lipider
- Blodprøvetagning til fedtsyresammensætning af membran fosfolipider i RBC
- Blodprøvetagning til adipokiner og cytokiner
- Blodprøvetagning til metabolisk profilering
- Måling af blodtryk efter 10 minutters hvile i siddende stilling
- Aflevering af 24 timers urinopsamling
- Helkrops DEXA-scanning + sagittal-højde (måling af kropssammensætning og abdominal fedme)
- OGTT med glukose-, c-peptid- og insulinmåling mv. fastende og over 3 timer. Spoturin udtages før og efter testen til metabolisk profilering.
- Afmontering af HOLTER-apparat til måling af Hjerte Rytme Variabilitet (HRV)
- Fedtvævsbiopsi
- MR skanning

7.1.7. Besøg 7

Besøg 7 skal finde sted 2 uger ± 5 dage efter besøg 6.

Følgende vil blive foretaget:

- Vægttabsvejledning/kostinstruktion ved diætist
- Måling af kropsvægt
- Registrering af medicinforbrug
- Registrering af uønskede hændelser

7.1.8. Besøg 8

Besøg 8 skal finde sted 4 uger ± 5 dage efter besøg 6.

Følgende vil blive foretaget:

- Vægttabsvejledning/kostinstruktion ved diætist
- Måling af kropsvægt
- Registrering af medicinforbrug
- Registrering af uønskede hændelser

7.1.9. Besøg 9

Besøg 9 skal finde sted 6 uger ± 5 dage efter besøg 6.

Følgende vil blive foretaget:

- Vægttabsvejledning/kostinstruktion ved diætist
- Måling af kropsvægt
- Registrering af medicinforbrug
- Registrering af uønskede hændelser

7.1.10. Besøg 10

Besøg 10 skal finde sted 8 uger ± 5 dage efter besøg 6.

Følgende vil blive foretaget:

- Vægttabsvejledning/kostinstruktion ved diætist
- Måling af kropsvægt
- Registrering af medicinforbrug
- Registrering af uønskede hændelser

7.1.11. Besøg 11

Besøg 11 skal finde sted 10 uger ± 5 dage efter besøg 6.

Følgende vil blive foretaget:

- Vægttabsvejledning/kostinstruktion ved diætist
- Måling af kropsvægt
- Registrering af medicinforbrug
- Registrering af uønskede hændelser
- Instruktion i 3-dages registrering af fysisk aktivitet i dagene op til besøg 12
- Instruktion i 3-dages vejet kostregistrering i dagene op til besøg 12

7.1.12. Besøg 12: Follow-up

Besøg 12 skal finde sted 12 uger ± 7 dage efter besøg 6. Besøg 12 vil strække sig over 2 dage:

Følgende vil blive foretaget dag 1:

- Påbegyndelse af 24 timers urinopsamling
- Påsætning af HOLTER-apparat til måling af Hjerte Rytme Variabilitet (HRV)

Følgende vil blive foretaget dag 2:

- Registrering af medicinforbrug
- Registrering af uønskede hændelser
- Måling af kropsvægt
- Måling af talje- og hofteomkreds
- Blodprøvetagning til hæmatologi
- Blodprøvetagning til biokemi
- Blodprøvetagning til lipider
- Blodprøvetagning til fedtsyresammensætning af membran fosfolipider i RBC
- Blodprøvetagning til adipokiner og cytokiner
- Blodprøvetagning til metabolisk profilering
- Måling af blodtryk efter 10 minutters hvile i siddende stilling
- Aflevering af 24 timers urinopsamling
- Helkrops DEXA-scanning + sagittal-højde (måling af kropssammensætning og abdominal fedme)
- OGTT med glukose-, c-peptid- og insulinmåling mv. fastende og over 3 timer. Spoturin udtages før og efter testen til metabolisk profilering.
- Afmontering af HOLTER-apparat til måling af Hjerte Rytme Variabilitet (HRV)
- Vægttabs-vejledning/kostinstruktion ved diætist (vil evt. foregå ved separat besøg)
- Fedtvævsbiopsi
- MR skanning

7.2. Stratificering

Ved baseline vil forsøgspersonerne blive blokrandomiserede (med hensyn til taljeomkreds) til forsøgsarmene og et randomiseringsnummer vil blive tildelt. Cut-off værdien, med hvilken blokkene vil blive inddelt, er 85 cm.

7.3. Metoder, primære effektparametre

7.3.1. Blodlipider

Der foretages målinger i fastende tilstand af indhold af serum-totalkolesterol, serum-triglycerider, serum-HDL-kolesterol samt serum-LDL-kolesterol. Undersøgelsen foretages på samme blod, som udtages til biokemi.

7.3.2. Glukosetolerans

Der foretages oral glukose tolerans test (OGTT). Hver forsøgsperson vil indtage 75 g glukose opløst i 300 ml vand indenfor en 2 minutters periode. Forsøgspersonerne vil forblive liggende og i hvile igennem proceduren, og skal faste (bortset fra vand) indtil testen er gennemført. Blodprøver vil blive taget til analyse af glukose, insulin og C-peptid (heparin-glas) til tidspunkterne (i alt 13 prøver): -10, 0, 10, 20, 30, 45, 60, 75, 90, 105, 120, 150 og 180 min i forhold til glukoseindtag.

ISI(composite) beregnes som 10.000/√[(fasteglukose(mg/dl) x fasteinsulin(µU/ml)) x (middel-glukose x middel-insulin under OGTT)] (25).

ISI(HOMA) beregnes som [fasteinsulin (µU/ml) x fasteglukose (mmol/l)]/22.5.

Andre aspekter af metabolisme under OGTT vil endvidere kunne undersøges via analyse af blodprøverne for proinsulin, GLP-1, GIP, FFA, glycerol, triglycerid (EDTA-glas) IGF-1og væksthormon (tørglas) m.fl.

7.3.3. Fedtaflejring i leveren

Til bestemmelse af fedtaflejring i leveren udføres magnetisk resonans spektroskopi (MRS)-skanninger på Herlev Hospital, Radiologisk Afdeling.

7.4. Metoder, sekundære effektparametre

7.4.1. Vægt, højde, talje- og hofteomkreds og sagittal højde

Vægt vil blive målt ved hvert besøg. Forsøgspersonerne vejes i deres undertøj efter at have været på toilettet og på en elektronisk vægt, resultater angives til nærmeste 0,1 kg.

Højde vil blive målt en gang i forbindelse med screening, afrundet til nærmeste 0,5 cm, når forsøgspersonen står op imod et vægtmonteret stadiometer med samlede fødder uden sko og hovedet i horisontalt niveau.

Taljeomkreds vil blive målt til nærmeste 0,5 cm, når forsøgspersonerne står op, ved brug af et målebånd. Taljeomkreds måles midt mellem hoftebensranden og brystkassen. Hofteomkredsen vil blive målt som den største omkreds i området omkring numsen [IHE standard SOP].

Sagittal højde måles, når patienten hviler sig i liggende stilling på et undersøgelsesbord, som afstanden mellem toppen af undersøgelsesbordet og en horisontalt placeret libelle placeret over abdomen på niveau med hoftebensranden i udåndingsfase. Forsøgspersonen bør ikke være iklædt stramtsiddende tøj [IHE standard SOP].

7.4.2. DEXA

Kropssammensætning måles ved helkrops dual-energy X-ray absorption (DEXA) scanning ved brug af et DPX dual-energy X-ray absorptiometer. Fedtmasse og fedtfri masse beregnes [IHE standard SOP].

Alle scanninger vil blive udført med en passende scanningshastighed alt efter kropsvægt og sagittal-højde. Varigheden af scanningen forventes at være 5-10 min. Forsøgspersonen vil modtage 0.04 – 0.08 mrem stråling pr. scanning. Alle forsøgspersoner vil blive scannet i fastende tilstand og kun iklædt let beklædning. Det totale scanningsområde vil måle 61 cm x 196 cm. Forsøgspersonen vil blive omviklet et bredt bælte, lavet af groft tætvævet stof, der lukkes ved hjælp af velcro-stropper for at fastholde forsøgspersonens position under scanningen.

Den samme person vil være scannings-analysator igennem hele forsøget, for at undgå forskel i analyser af scanningsresultaterne. Kvalitetskontrol og kalibreringskontrol vil blive udført mindst en gang om ugen med anvendelse af Lunar Aluminum Spine Phantom (LSP).

7.4.3. Hjerte Rytme Variabilitet (HRV)

HRV måles ved at forsøgsdeltageren møder om morgenen og får påsat en digital Holter monitor, som bæres i mavebælte (på størrelse med en walkman). Optageren forsynes med et digitalt hukommelseskort med henblik på lagring af hjertets elektriske impulser gennem 24 timer. Fire elektroder påsættes deltagerens brystkasse og tilsluttes optageren. Før deltageren forlader stedet sikres det via et konventionelt EKG-apparat, at der opnås sufficient elektrisk signal. Deltageren bærer optageren i 24 timer og møder næste morgen, hvor optageren afmonteres og kortet udtages.

7.4.4. Fedtvævsbiopsi

1-2 g fedtvævsprøve vil blive udtaget fra abdominalt subcutant fedtdepot under lokalbedøvelse. Biopsierne vil blive renset for blod med sterilt saltvand og øjeblikkeligt nedfrosset med flydende nitrogen. Opbevares ved -80 ºC. I fedtvævsbiopsier analyseres fedtsyresammensætning af triglycerider (rør 1), indhold af ceramid (rør 1), samt ekspression (mRNA) af cytokiner (rør 2). Desuden udføres magic angle spinning NMR analyser (rør 3).

7.4.5. Inflammatoriske markører og adiponektin

Relevante inflammatoriske markører, cytokiner og adipokiner associeret med dyslipidæmi og insulinvirkning som hypersensitiv CRP, TNF-α, IL-6, IL-8 og adiponektin vil blive målt i plasma (protein-niveau).

Desuden vil gen-ekspression (mRNA) af cytokiner blive bestemt i fedtvæv (se afsnit 7.4.4).

7.4.6. Metabolisk profilering

Både på fasteblodprøver (5 ml blod i citratglas) og på plasmaprøverne udtaget i forbindelse med OGTT foretages metabolisk profilering (metabolomics) med NMR og med massespektrometri (LC-MS). Dette følges op med identifikation af metabolitter, der i multivariat analyse viser forskelle mellem prøverne fra de forskellige behandlinger. Der vil blive gennemført proteinprofilering (proteomics) på plasmaprøverne samt transkriptomanalyser på RNA fra leucocytter. Desuden isoleres erythrocytter, hvilket giver mulighed for at isolere membranfraktionen til NMR-baseret lipidomics samt cytosolen for analyse af antioxidativt forsvar.

Døgnurin opsamles for analyse af biomarkører for indtag af transfedtsyrer. Para-amino benzoesyre (PABA), (i alt 240 mg pr. urinopsamlingsdag) anvendes som markør for fuldstændighed af opsamling. Der foretages metabolisk profilering med NMR og med massespektrometri på urinprøverne, samt på spoturinprøver taget før og efter OGTT, som følges op med identifikation af metabolitter, der i multivariat analyse viser forskelle mellem prøverne fra de forskellige behandlinger.

Desuden udføres magic angle spinning NMR analyser på fedtvæv.

7.4.7. MR scanning

Til bestemmelse af mængden af visceralt fedt og intramuskulært fedt udføres MR-skanninger på Herlev Hospital, Radiologisk Afdeling.

7.5. Metoder, sikkerhedsparametre

Forsøget og forsøgsprocedurerne forventes ikke at resultere i nogen uønskede hændelser, men som ved ethvert forskningsforsøg, er der mulighed for uforudsete risici, som ikke kan forudses hverken af forskerne eller deltagere.

I alle kliniske undersøgelser bør der udføres en sikkerhedsprofil og der er her valgt en hæmatologiprofil, en biokemiprofil samt monitorering af blodtryk.

7.5.1. Hæmatologi

Der tages 5 ml blod fra en cubital vene i Kalium-EDTA glas. Glasset vendes forsigtigt 5 til 10 gange efter prøvetagningen og sendes samme dag til Klinisk-biokemisk afdeling, Amtssygehuset i Gentofte, for analyse.

Følgende analyser udføres: Hæmoglobin, thrombocyttal , leukocyttal, erythrocyttal.

7.5.2. Biokemi

Der tages 2 x 10 ml blod i gelglas fra en cubital vene. Glasset henstilles til koagulering ved stuetemperatur i minimalt 30 og maksimalt 60 minutter, hvorefter det slynges ved 3.000 omdrejninger pr. min og sendes til Klinisk-biokemisk afdeling, Amtssygehuset i Gentofte. Der foretages følgende undersøgelser: creatinin, albumin, bilirubin, alanin-aminotransferase (ALAT), aspartataminotransferase (ASAT) alkalisk fosfatase, kalium og natrium.

Restserum opbevares ved en temperatur under minus 70 grader på Klinisk-biokemisk afdeling, Amtssygehuset i Gentofte, for analyse af eventuelle andre komponenter efter studieophør.

7.5.3. Blodtryk og puls

Blodtryk vil blive målt efter forsøgspersonen har hvilet sig i siddende stilling i 10 minutter. Der foretages 3 målinger af blodtrykket og gennemsnittet af de to sidste målinger vil blive indført i CRF'en. Puls for første måling vil blive indført i CRF'en [IHE standard SOP].

7.6. Metoder, kontrol og bestemmelse af compliance

7.6.1. Kostvejledning/instruktion

Kostvejledning vil blive givet ved alle besøg på nær ved screening. Under kostinterventionsperioden noterer forsøgspersonerne i en udleveret dagbog, hvor meget af den udleverede forsøgskost de indtager. Ved det efterfølgende vægttabsprogram anvendes dagbøger til registrering af dagligt kostindtag i samråd med en diætist.

7.6.2. 3-dages kostregistrering

Ved screening, i slutningen af kostinterventionsperioden og ved opfølgningsbesøget vil forsøgspersonerne blive instrueret i at registrere kost i en kostdagbog i 3 dage En af dagene bør være en dag i weekenden. Forsøgspersonerne bør ikke lade registreringen af mad influere på deres daglige kost. Instruktion i kostregistreringerne vil blive givet af en diætist.

7.6.3. 3-dages registrering af fysisk aktivitet

Ved screening, i slutningen af kostinterventionsperioden og ved opfølgningsbesøget vil fysisk aktivitet blive vurderet over 3 dage hos alle forsøgsdeltagere ved hjælp af et skema, hvorpå graden af fysisk aktivitet skal angives hvert kvarter (se Appendiks 7: Skema for registrering af fysisk aktivitet). Herudfra kan deltagernes energiforbrug beregnes (26).

7.6.4. Compliance

Compliance over for interventionskosten vurderes ved måling af fedtsyrer i cellulære compartments. Som måling for en længere periode (~2-8 uger) anvendes fedtsyresammensætning i fosfolipider i de røde blodlegemers cellemembraner. Til bestemmelse af dette udtages 10 ml K-EDTA blod.

Et godt udtryk for personens indtag over perioder >6 måneder fås ved måling af fedtsyremønstret i en fedtvævsbiopsi, men indtaget kan muligvis detekteres tidligere. Abdominal fedtvævsbiopsi udtages med kanyle efter forudgående indgivelse af lokal anæstesi.

7.7. Håndtering af biologisk materiale

Al udtaget biologisk materiale vil blive opbevaret i personhenførbar form til og med år 2011, hvorefter alt materiale vil blive anonymiseret. Materialet vil blive opbevaret på Institut for Human Ernæring, eller på de institutioner hvortil forskningsprojektets partnere er affilieret (se afsnit 18. Partnere).

8. Interventionskost

Forsøget udføres som et substitutionsforsøg, idet der udleveres kost, som erstatter forsøgspersonens indtag af anden mad. Der er valgt et tilskud af IP-TFA i en fedtblanding svarende til 15 g transfedtsyrer pr. dag (25 g transfedtsyreberiget fedt idet TFA udgør 60% af fedtet).

Til kontrolgruppen udleveres en kost, hvor 25 g kontrolfedt (med 60% oliesyre) erstatter forsøgspersonens indtag af anden mad.

8.1. Industrielt produceret transfedtsyreberiget kost

I samarbejde med AarhusKarlshamn Denmark A/S fremstilles testfedt indeholdende 60% hyppigt forekommende isomerer af transfedtsyrer (18:1 og 18:2 *trans*). Det fremstillede testfedt inkorporeres i en mængde svarende til 25 g pr. dag i boller.

8.2. Kontrolkost

Fra AarhusKarlshamn Denmark A/S modtages kontrolfedt indeholdende ca. 60% oliesyre. Kontrolfedt inkorporeres i en mængde svarende til 25 g pr. dag i boller.

8.3. Opbevaring af forsøgsmateriale

Testfedtet leveres i tætsluttende emballage af passende størrelse. Testfedt opbevares på køl.

9. Registrering af sygdom og medicinindtag

9.1. Baseline

Tilstedeværende sygdomme samt al brug af medicin, dels som fast medicinering dels anvendt inden for de sidste 3 måneder før inklusion, registreres.

9.2. Forsøgsforløb

Under alle studiebesøg registreres eventuelle opståede sygdomme samt medicinindtag.

10. Bivirkninger

Der forventes ikke ved et kosttilskud med transfedtsyrer eller oliesyre nogen bivirkning ud over eventuelle lette gastrointestinale, som abdominalt ubehag og eventuel løs afføring/diarre.

10.1. Definitioner

10.1.1. Bivirkninger

En bivirkning er enhver uønsket medicinsk hændelse, som indtræffer hos en forsøgsperson i et klinisk forsøg. Dette er uafhængigt af om det betragtes som relateret til forsøget eller de anvendte forsøgsprodukter. Bivirkninger inkluderer begivenheder, som ikke er registreret ved baseline eller som er forværret ved det pågældende besøg i forhold til baseline. En bivirkning er således ikke en tilstand, der er registreret og som tillod personen at blive inkluderet, og som ikke forværres i studieforløbet.

10.1.2. Laboratorie - "bivirkninger"

En "laboratoriebivirkning" er enhver abnormitet i de laboratoriemæssige fund, som den studieansvarlige finder af en sådan sværhedsgrad, at det nødvendiggør klinisk stillingtagen.

10.1.3. Alvorlige bivirkninger

Enhver hændelse udover de ovenfor anførte som af den studieansvarlige vurderes af klinisk betydning.

10.2. Rapportering af bivirkninger

Alle oven for nævnte bivirkninger skal indføres på patientens CRF.

11. Case Report Forms (CRF)

Case Report Forms (CRF) til optegnelse af data der indhentes fra forsøgspersoner, der deltager i studiet, vil blive fremstillet af den protokolansvarlige på IHE.

11.1. Regler for brug af CRF

Udfyldes tydeligt med kuglepen. Alle relevante spørgsmål skal besvares.

Hvis en undersøgelse/prøve ikke er udført, og derfor ikke vil være tilgængelig i studiet skal dette anføres med ND (Not Done) i de respektive felter på CRF.

11.2. Rettelser i CRF

Rettelser i CRF kan kun foretages ved at udstrege (med kuglepen) de ukorrekte data, således at de fortsat er læselige og derefter anføre den korrekte værdi ved siden af den overstregede med signatur og datering. I givet fald ledsaget af en skriftlig forklaring på ændringen/korrektionen.

12. Databehandling

12.1. Kvalitetssikring af data

Alle kildedokumenter vil indgå som studiedata. Data registreret i disse dokumenter vil blive indsamlet i overensstemmelse med god klinisk praksis (GCP).

12.2. Statistiske metoder

Alle analyser og tabuleringer vil blive udført ved hjælp af de seneste udgaver af SAS® eller SPSS software eller lignende statistisk software. Statistiske tests vil blive udført ved at anvende 5% som det nominale signifikansniveau og intervalestimater vil blive bestemt ved 95% konfidensinterval.

Statistiske og analytiske metoder og overvejelser om prøvestørrelse diskuteres i de følgende sektioner.

12.2.1. Formål med analyser

At bestemme effekterne af en kost med højt indhold af IP-TFA sammenlignet med en kontrolkost, hvor TFA er erstattet af oliesyre.

12.2.2. Primære effektmål

De primære effektmål er ændring i leverfedtmasse, i insulinvirkning beregnet som ISI(composite) under oral glukose tolerans test samt i lipidæmi beregnet som LDL-kolesterol/HDL-kolesterol ratio.

Associerede mål for dyslipidæmi er ændring i TC, TC/HDL-kolesterol-ratio, HDL-kolesterol, LDL-kolesterol samt triglycerid. Associerede mål for insulin resistens er ISI(HOMA), faste glukose, faste insulin og glukose tolerans.

12.2.3. Sekundære effektmål

Sekundære effektmål er talje, hofte og sagittalmåling, abdominal fedtmasse, intramuskulært fedt, inflammatoriske markører, hjerterytmevariabilitet, ekspression af cytokiner i fedtvæv, og ceramid i fedtvæv.

Andre mål i studiet er at identificere eventuelle sammenhænge mellem inkorporering af transfedtsyrer i RBC membranlipider samt i fedtvæv og de nævnte effektmål.

Metabolisk profilering vil blive gennemført på fedt-, urin- og plasmaprøver ved hjælp af NMR-analyse og på urin og plasma med LC-MS.

12.2.4. Statistisk metode for de primære effektparametre

Ændringer i de kontinuerte variable fra baseline til uge 16 og uge 28 indenfor grupper vil blive testet ved kovarians analyse ved at anvende baselineværdier som kovariater og ved justering for stratificeringsvariablen taljemål, hvor det skønnes relevant. Alle nul-hypoteser opsat med det formål at sammenligne grupper vil antage ens effekt mellem grupperne, mens den alternative hypotese antager, at der er en forskel i effekt (to-sidet analyse).

12.2.5. Statistiske metoder for sekundære effektparametre

Kovarians-analyse vil blive benyttet til at sammenligne de sekundære effektparametre ved uge 16 og uge 28 mellem grupper, og ved at benytte relevante data fra baseline som kovariater og ved justering for stratificeringsvariablen taljemål, hvor det skønnes relevant.

Data fra metabolisk profilering vil blive analyseret mod de øvrige effektvariable ved hjælp af kemometri, herunder multivariate analyser under anvendelse af bl.a. Matlab, Latentix og anden matematisk/statistisk analytisk software. Resultaterne herfra vil generere post hoc hypoteser om nye biomarkører for eksponering eller effekt. Det kan dreje sig om bestemte metabolitter, proteiner eller genekspression, der herefter vil blive målt separat og analyseret statistisk på samme måde som andre effektvariable.

12.2.6. Estimering af prøvestørrelse

Da der ikke foreligger tidligere studier af transfedtsyrers virkning på glukosestofskifte og aflejring af fedt i leveren, er det ikke muligt at gøre begrundede antagelser med henblik på en styrkeberegning for disse endepunkter. Man da påvirkning af disse variable må forventes at optræde sammen med påvirkning af blodets lipoproteiner, som er velbeskrevet, anvendes ændringer heri til beregningen.

Prøvestørrelsen for studiet er følgelig valgt med det formål at give den nødvendige styrke (power) til at detektere forskelle på ændring i LDL-kolesterol/HDL-kolesterol ratio, som skyldes effekten af de forskellige kosttyper indtaget over 16 uger. I nulhypotesen antager vi, at de to interventionsgrupper ved uge 16 har samme LDL-kolesterol/HDL-kolesterol ratio sammenlignet med baseline, mens den alternative hypotese antager, at der er forskel i LDL-kolesterol/HDL-kolesterol ratio mellem behandlingsgrupperne ved uge 16.

Forskellen i LDL-kolesterol/HDL-kolesterol ratio efter længere tids indtagelse af kosttyper med hhv. 7 E% IP-TFA eller tilsvarende cis-umættede fedtsyrer er i to metaanalyser fundet at være 0,4-0,5 mmol/l (5;6) .

Idet forsøgspersonerne i det planlagte studie er præobese, så antager vi at kunne observere en forskel på 0,5 mmol/l i LDL-kolesterol/HDL-kolesterol ratio efter 16 ugers kostintervention og vi antager en SD på 0,6 mmol/l som fundet i et tidligere studie (27).

Ved antagelsen af en forskel mellem kontrolfedt- og transfedtgruppen på 0,5 mmol/l (SD 0,6 mmol/l), og mindst 80% statistisk power og et signifikans niveau på 5%, så behøves ca. 24 deltagere pr. kostgruppe (uparret t-test).

Eftersom de fleste andre studie-variable er sekundære effektparametre, er der ikke udført beregninger af prøvestørrelse af andre variable end den primære effektparameter. Mht. de endelige resultater, så accepteres det derfor, at post-hoc analyser kan eller vil finde, at ikke alle målinger af primære og sekundære effektparametre nødvendigvis har/havde den tilstrækkelige prøvestørrelse.

13. Etik

13.1. Videnskabsetisk komité bedømmelse

Inden studiet påbegyndes, indsender den forsøgsansvarlige studieprotokollen med tilhørende dokumentation til den lokale/regionale Videnskabsetiske Komité. Studiet må ikke iværksættes inden protokollen er godkendt af den lokale/regionale Videnskabsetiske Komité. Godkendelse skal indhentes i skriftlig form og skrivelsen skal opbevares på stedet, hvor studiet gennemføres.

Den Videnskabsetiske Komité skal konsulteres, hvis omstændigheder gør, at der skal indføres betydelige ændringer til protokollen eller bilag til denne i løbet af studiet.

13.2. Etisk gennemførelse af studiet

Studiet gennemføres i overensstemmelse med de relevante internationale konventioner og deklarationer, inklusiv de seneste udgaver af:

- Helsinki Deklarationen.
- Lov nr. 402 af 28. maj 2003 om et videnskabsetisk komitésystem og behandling af biomedicinske forskningsprojekter (Komitéloven)
- Vejledning om anmeldelse m.v. af et biomedicinsk forskningsprojekt til det videnskabsetiske komitésystem
- Bekendtgørelse nr. 806 af 12. juli 2004 om information og samtykke ved inddragelse af forsøgspersoner i biomedicinske forskningsprojekter

Forsøgspersoner er forsikret gennem den offentlige patientforsikring.

Studiet er anmeldt til Datatilsynet via en fællesanmeldelse for Institut for Human Ernæring, journalnummer 2002-54-0938.

13.3. Mulige fordele af studiet og sikkerhedsaspekter

Fordelene for forsøgspersonerne, og for samfundet som helhed, opvejer eventuelle risici til fulde. COBRA-studiet har potentiale til at præge fremtidig forskning angående effekter af transfedt i kosten. Formålet med studiet er ikke at behandle en sygdom, men forsøgspersonerne i dette projekt forventes alligevel at modtage umiddelbare fordele ved deltagelse i studiet i form af gentagne målinger og monitoreringer af adskillige sundhedsrelaterede faktorer, herunder BMI, blodlipider, blodtryk og kropssammensætning.

Information om effekten af indtag af transfedtsyrer, som kan fremkomme af dette studie, kan hjælpe de fremtidige offentlige anbefalinger og politikken inden for regulering af IP-TFA-indholdet i fødevarer. En sådan regulering vil potentielt kunne forebygge udvikling af en række hastigt stigende sygdomme i befolkningen, herunder metabolisk syndrom, type 2 diabetes og hjertekarsygdom.

Det ubehag/de gener som studiets deltagere måtte opleve, antages i alle tilfælde at være milde og forbigående. De risici, som forventeligt vil opstå i studiet, vil være begrænset til mindre ubehag i forbindelse med blodprøvetagning/injektioner, og i forbindelse med udtagning af fedtbiopsier, hvor der kan opstå mindre blodansamlinger ved prøveudtagningsstedet. De potentielle risici forbundet med de benyttede kostelementer i de respektive kostgrupper mht. udvikling af hjertekarsygdom og type 2 diabetes vil blive grundigt undersøgt i løbet af studiet.

Kostændringerne i forsøget (15 g/d transfedtsyrer) svarer til, hvad der almindeligt forekommer i vestlige befolkninger med hensyn til fødevalg (28). De i forsøget eventuelle forårsagede ændringer i effektmål (fedtstoffer i blodet, insulinfølsomhed og kropssammensætning mm.) i såvel positiv som negativ retning er forbigående og vil, efter vor bedste overbevisning, ikke medføre nogen ændring i deltagernes senere risiko for at udvikle hjertesygdom eller type 2 diabetes. De ændringer, der i et tidligere studie observeredes efter 8-ugers kostintervention med 20 g/d transfedt var reverseret til baselineværdier ved et opfølgningsbesøg 12 uger efter interventionens afslutning (9).

Desuden vil alle forsøgsdeltagere umiddelbart efter kostinterventionen deltage i et vægttabsprogram under diætistvejledning, efter hvilken der, for at undersøge reversibilitet, foretages en efterundersøgelse af alle effektparametre. Dette vægttabsprogram har potentiale til varigt at ændre forsøgspersonernes kost- og livsstils-vaner i en helbredsmæssig gunstig retning.

13.4. Samtykkeerklæring

Informeret samtykke skal gives frivilligt af hver enkelt forsøgsperson, efter at de er blevet fuldt informeret både mundtligt og skriftligt om studiet, om indholdet og formålet med studiet, effekter og mulige bivirkninger og risici ved deltagelse i studiet.

Forsøgspersonen skal orienteres om, at hun til enhver tid kan trække sit samtykke tilbage og udtræde af forsøget. Forsøgspersonen skal have besked på, at dennes deltagelse i studiet er fortrolig, men skal også vide, at oplysninger kan videregives til relevante offentlige myndigheder, såsom videnskabsetisk komité. Såvel forsøgspersonen som forskeren underskriver samtykkeerklæring. Der henvises til Appendiks 5: Samtykkeerklæring.

13.5. Forsøgspersoners privatliv

Forskeren bekræfter og opretholder principperne for forsøgspersonens rettigheder til beskyttelse af privatlivet. Alle kildedokumenter og andre studierelevante dokumenter skal behandles som fortrolige og identificeres ved behandlingsnummer, sagsmappenummer, personinitialer og fødselsdato. Forskeren skal betragte al information som fortrolig og må ikke afsløre det, medmindre loven kræver det.

14. Hvervning af deltagere

Forsøgspersoner hverves via annoncer publiceret i lokaler aviser. Deltagere i hovedstudiet og referencedeltagere vil blive hvervet ved to forskellige annoncer (Appendiks 6a: Annoncetekst til hvervning af deltagere og Appendiks 2b. Deltagerinformation, normalvægtige reference). Ved respons på annonce, vil den skriftlige deltagerinformation blive fremsendt enten via e-mail eller almindelig post (Appendiks 2a: Deltagerinformation eller Appendiks 2b. Deltagerinformation, normalvægtige reference) sammen med en folder, der beskriver forsøgspersonens rettigheder (Appendiks 3: ”Dine rettigheder som forsøgsperson i et biomedicinsk forskningsprojekt”). Samtidig inviteres personen ifølge med en evt. bisidder til et informationsmøde på Institut for Human Ernæring (Appendiks 4: Procedure for afgivelse af mundtlig deltagerinformation). Her vil den skriftlige deltagerinformation også være tilgængelig.

15. Tilgængelighed af oplysninger for forsøgspersoner

Forsøgspersonerne kan efter ønske få adgang til at se alle papirerne vedrørende selve forsøget, bortset fra de dele, der indeholder private oplysninger om de andre deltagere. Dette kan ske ved at rette henvendelse til den daglige forsøgskoordinator og kontaktperson Nathalie Tommerup Bendsen. Undersøgelsesresultater vil, efter ønske, blive givet til den enkelte forsøgsdeltager ved afslutning af dataindsamling og -behandling, såfremt forsøgspersonen har ytret ønske herom på Samtykkeerklæringen (se Appendiks 5: Samtykkeerklæring).

16. Offentliggørelse af forsøgsresultater

Studiet registreres på www.clinicaltrials.gov

Arne Astrup er den primære ansvarlige for videnskabelige publikationer. Forfatterskab vil blive inkluderet i henhold til Vancouver Deklarationen. Såvel positive som negative forsøgsresultater vil blive offentliggjort. Rapporter vil blive publiceret i relevante internationale og nationale videnskabelige peer-reviewed tidsskrifter, ikke-videnskabelige tidsskrifter eller andre relevante medier.

17. Økonomiske forhold

Forskningsprojektet er initieret af den forsøgsansvarlige Arne Astrup, Institut for Human Ernæring. Den økonomisk ansvarlige er Institutleder Arne Astrup. Studiet har modtaget kr. 2.072.000 i støtte fra Det Strategiske Forskningsråd, Programkomitéen for Fødevarer og Sundhed (FøSu). Pengene udbetales til

Institut for Human Ernæring over en fireårig periode fra 2008-2011 og går til aflønning af TAP, betaling af laboratorieundersøgelser og andre driftsudgifter. Studiet har endvidere modtager kr. 58.000 i støtte fra Diabetesforeningen. Pengene udbetales til Institut for Human Ernæring i 2008 og går til analyser af blodprøver. Den forsøgsansvarlige har ingen økonomisk tilknytning til støttegivere. Studiet søges i øvrigt støttet af såvel offentlige som private fonde.

Der udbetales honorar på 3000 kr. (beskattet som A-indkomst) til hver forsøgsdeltager i én af interventionsgrupperne efter endt gennemførelse af besøg 1-6. I tillæg hertil udbetales 1500 kr (beskattet som A-indkomst) i ulempegodtgørelse til de forsøgsdeltagere, der gennemfører opfølgningsbesøget efter vægttabsforløbet.

Der udbetales honorar på 2500 kr. (beskattet som A-indkomst) til hver forsøgsdeltager, som inkluderes i referencegruppen, efter endt gennemførelse af besøg 1 og 2.

18. Partnere

Forskningsprojektet udføres som et samarbejde mellem forsøgets hovedansvarlige Arne Astrup, institutleder, professor, dr. med., Institut for Human Ernæring, Det Biovidenskabelige Fakultet, Københavns Universitet og

- Nathalie Tommerup Bendsen, PhD studerende, Institut for Human Ernæring, Det Biovidenskabelige Fakultet, Københavns Universitet
- Thomas Meinert Larsen, adjunkt, PhD, Institut for Human Ernæring, Det Biovidenskabelige Fakultet, Københavns Universitet
- Jørn Dyerberg, professor (emeritus) dr. med., Institut for Human Ernæring, Det Biovidenskabelige Fakultet, Københavns Universitet
- Steen Stender, overlæge dr. med., Klinisk-biokemisk Afdeling, Gentofte Amtssygehus
- Steen B. Haugaard, 1. res.læge, dr.med., Klinisk forskningsenhed, Hvidovre Hospital, Københavns Universitet
- Steen Bønløkke Pedersen, overlæge, forskningslektor, dr.med. Endokrinologisk Afdeling, THG, Århus Sygehus, Århus Universitet
- Huiling Mu, lektor, PhD, Biocentrum, Danmarks Tekniske Universitet
- Lars Ove Dragsted, professor, Institut for Human Ernæring, Det Biovidenskabelige Fakultet, Københavns Universitet

Ingen af forsøgets partnere har økonomiske interesser i studiet.

19. Referencer

1. Oomen CM, Ocké MC, Feskens EJM, van Erp-Baart MJ, Kok FJ, Kromhout D. Association between trans fatty acid intake and 10-year risk of coronary heart disease in the Zutphen Elderly Study: a prospective polulation-based study. Lancet 2001;357:746-51.

2. Hu FB, Stampfer MJ, Manson JE et al. Dietary Fat Intake and the Risk of Coronary Heart Disease in Women. The New England Journal of Medicine 1997;337:1491-9.

3. Salmeron J, Hu FB, Manson JE et al. Dietary fat intake and risk of type 2 diabetes in women. Am J Clin Nutr 2001;73:1019-26.

4. Sun Q, Ma J, Campos H et al. A prospective study of trans fatty acids in erythrocytes and risk of coronary heart disease. Circulation 2007;115:1858-65.

5. Mozaffarian D, Katan MB, Ascherio A, Stampfer MJ, Willett WC. Trans Fatty Acids and Cardiovascular Disease. The New England Journal of Medicine 2006;354:1601-13.

6. Ascherio A, Katan MB, Zock PL, Stampfer MJ, Willett WC. Trans Fatty Acids and Coronary Heart Disease. The New England Journal of Medicine 1999;340:1994-8.

7. Breslow JL. n-3 Fatty acids and cardiovascular disease. Am J Clin Nutr 2006;83:S1477-S1482.

8. Christensen JH, Christensen MS, Dyerberg J, Schmidt EB. Heart rate variability and fatty acid content of blood cell membranes: a dose-response study with n-3 fatty acids. Am J Clin Nutr 1999;70:331-7.

9. Dyerberg J, Eskesen DC, Andersen PW et al. Effects of trans- and n-3 unsaturated fatty acids on cardiovascular risk markers in healthy males. An 8 weeks dietary intervention study. European Journal of Clinical Nutrition 2004;58:1062-70.

10. Kavanagh K, Jones KL, Sawyer J et al. Trans Fat Diet Induces Abdominal Obesity and Changes in Insulin Sensitivity in Monkeys. Obesity 2007;15:1675-84.

11. Koh-Banerjee P, Chu NF, Spiegelman D et al. Prospective study of the association of changes in dietary intake, physical activity, alcohol consumption, and smoking with 9-y gain in waist circumference among 16 587 US men. Am J Clin Nutr 2003;78:719-27.

12. Thomas E, Hamilton G, Patel N et al. Hepatic triglyceride content and its relation to body adiposity: a magnetic resonance imaging and proton magnetic resonance spectroscopy study. Gut 2005;54:122-7.

13. Kelley DE, McKolanis TM, Hegazi RAF, Kuller LH, Kalhan SC. Fatty liver in type 2 diabetes mellitus: relation to regional adiposity, fatty acids, and insulin resistance. Am J Physiol Endocrinol Metab 2003;285:E906-E916.

14. Rader DJ. Effect of Insulin Resistance, Dyslipidemia, and Intra-abdominal Adiposity on the Development of Cardiovascular Disease and Diabetes Mellitus. The American Journal of Medicine 2007;120:S12-S18.

15. Kahn SE, Hull RL, Utzschneider KM. Mechanisms linking obesity to insulin resistance and type 2 diabetes. Nature 2006;444:840-6.

16. Lovejoy JC, Smith SR, Champagne CM et al. Effects of diets enriched in saturated (palmitic), monounsaturated (oleic), or trans (elaidic9 fatty acids on insulin sensitivity and substrate oxidation in healthy adults. Diabetes Care 2002;25:1283-8.

17. Louheranta AM, Turpeinen AK, Vidgren HM, Schwab US, Uusitupa MIJ. A high-trans fatty acid diet and insulin sensitivity in young healthy women. Metabolism 1999;48:870-5.

18. Christiansen E, Schnider S, Palmvig B, Tauber-Lassen E, Pedersen O. Intake of a diet high in trans monounsaturated fatty acids or saturated fatty acids. Effects on postprandial insulinemia and glycemia in obese patients with NIDDM. Diabetes Care 1997;20:881-7.

19. Lefevre M, Lovejoy JC, Smith SR et al. Comparison of the acute response to meals enriched with cis- or trans-fatty acids on glucose and lipids in overweight individuals with differing FABP2 genotypes. Metabolism 2005;54:1652-8.

20. Han SN, Leka LS, Lichtenstein AH, Ausman LM, Schaefer EJ, Meydani SN. Effect of hydrogenated and saturated, relative to polyunsaturated, fat on immune and inflammatory responses of adults with moderate hypercholesterolemia. J Lipid Res 2002;43:445-52.

21. Baer DJ, Judd JT, Clevidence BA, Tracy RP. Dietary fatty acids affect plasma markers of inflammation in healthy men fed controlled diets: a randomized crossover study. Am J Clin Nutr 2004;79:969-73.

22. Mozaffarian D, Pischon T, Hankinson SE et al. Dietary intake of trans fatty acids and systemic inflammation in women. Am J Clin Nutr 2004;79:606-12.

23. Mozaffarian D, Rimm E, King IB, Lawler RL, McDonald GB, Wayne CL. trans Fatty acids and systemic inflammation in heart failure. Am J Clin Nutr 2004;80:1521-5.

24. Kolak M, Westerbacka J, Velagapudi VR et al. Adipose Tissue Inflammation and Increased Ceramide Content Characterize Subjects With High Liver Fat Content Independent of Obesity. Diabetes 2007;56:1960-8.

25. Matsuda M, DeFronzo RA. Insulin sensitivity indices obtained from oral glucose tolerance testing - comparison with the euglycemic insulin clamp. Diabetes Care 1999;22:1462-70.

26. Bouchard C, Tremblay A, Leblanc C, Lortie G, Savard R, Thériault G. A method to assess energy expenditure in children and adults. Am J Clin Nutr 1983;37:461-7.

27. Aro A, Jauhiainen M, Partanen R, Salminen I, Mutanen M. Stearic acid, trans fatty acids, and dairy fat: effects on serum and lipoprotein lipids, apolipoproteins, lipoprotein(a), and lipid transfer proteins in healthy subjects. Am J Clin Nutr 1997;65:1419-26.

28. Stender S, Dyerberg J, Bysted A, Leth T, Astrup A. A trans world journey. Atherosclerosis Supplements 2006;7:47-52.

Appendiks 1: Lægmandsbeskrivelse

Ikke videnskabelig beskrivelse af studiet COBRA

Baggrund

Store befolkningsundersøgelser samt mekanismestudier tyder samstemmende på, at transfedtsyrer øger risiko for hjertekarsygdom. Transfedtsyrer dannes blandt andet ved industriel hærdning af spiseolier, og anvendes i margariner til brød og kager, i snacks, i fastfood, i slik og i mange andre madvarer. En analyse, hvor man samlet vurderede resultaterne fra en række store befolkningsundersøgelser, viste, at personer med en daglig indtagelse af transfedtsyrer svarende til 2% af kostens kalorieindhold, har en overdødelighed af hjertesygdomme på ca. 25%, sammenlignet med personer med et lavt indtag af transfedtsyrer.

Transfedtsyrer påvirker blodets kolesterolindhold i en retning som øger risikoen for åreforkalkning og for blodprop i hjertet. Undersøgelser på frivillige forsøgspersoner, som fik transfedtsyrer svarende til 2 % af kostens kalorieindhold, førte imidlertid kun til ændringer i blodets kolesterolindhold, som svarer til en forøgelse i risikoen for død af hjertesygdom på 5%. Der mangler således en yderligere forklaring på den skadelige virkning af transfedtsyrer, ud over deres uheldige virkning på blodets kolesterolindhold. Nyere studier peger på, at en del af effekten kan udspille sig gennem øget mavefedme, insulinresistens samt andre end de traditionelle risikofaktorer for hjertekarsygdom.

Der findes en række såkaldte risikomarkører for udvikling af hjertesygdom og sukkersyge, og dette muliggør, at man på raske forsøgspersoner kan udføre forsøg for at undersøge om disse risikomarkører kan påvirkes f.eks. af kosten. Forsøgspersonerne påføres ikke nogen risiko, idet forsøgene er kortvarige og fører til ændringer, som ved forsøgsophør, hurtig forsvinder. De behandler desuden forhold som afspejler almindelige kostvalg i befolkningen, men som undersøges under kontrollerede forhold.

Med dette studie ønsker vi, at belyse effekten af en kost med et højt indhold af industrielt fremstillede transfedtsyrer sammenlignet med en transfedtsyre-fri kost på mavefedme samt en række risikomarkører for udvikling af sukkersyge og hjertesygdom. Studiet vil desuden kunne afdække, hvordan en sådan effekt kan forklares. Endvidere ønsker vi at undersøge, hvad der sker, når man går fra en kost med et højt indhold af transfedt til en normal sund kost.

Forsøget COBRA

Navnet er et akronym for en engelsk titel: Copenhagen Obesity Risk Assessment study.

I forsøget indgår ca. 60 moderat overvægtige postmenopausale kvinder i alderen 45-70 år uden hjertesygdom, sukkersyge, forhøjet blodtryk eller andre alvorlige lidelser.

Efter et informationsmøde indkaldes deltagerne til en forundersøgelse, hvorefter deltagerne fordeles tilfældigt til 2 forsøgsgrupper.

Den ene gruppe får en del af kosten udskiftet med fødevarer (fortrinsvis brød), som fremstilles på Institut for Human Ernæring og som indeholder transfedtsyrer i en daglig mængde på 15 gram. Den anden gruppe er kontrolgruppen, som får en del af kosten udskiftet med fødevarer, der indeholder fedt med et højt indhold af oliesyre, som er en såkaldt *cis*-monoumættet fedtsyre.

Desuden rekrutteres 20 normalvægtige kvinder til at udgøre en referencegruppe, der kun undersøges én gang og dermed ikke skal gennemgå kostintervention.

Undersøgelser i forsøget

Selve kostperioden varer 16 uger, hvor deltagerne bliver indkaldt til undersøgelse i alt 5 gange: Ved kostperiodens start, og herefter med 4 ugers intervaller til dennes afslutning. Der tages blodprøver til

almindelige laboratorieprøver, der belyser leverfunktionen, nyrefunktionen osv. Prøverne svarer til, hvad man generelt vil betegne som et "helbredscheck". Derudover tages blodprøver til bestemmelse af blodets fedtstoffer, samt prøver der bl.a. afspejler betændelsesreaktioner mv.

De for studiet væsentlige undersøgelser er: Måling af blodets fedtstoffer, måling af sukkerstofskiftet og insulinfølsomheden samt mængden af fedt i leveren.

Sukkerstofskiftet og insulinfølsomheden undersøges før og efter kostperioden ved en såkaldt oral glukose tolerans test (OGTT). Forsøgspersonerne indtager 75 g druesukker opløst i vand, og der udtages blodprøver til bestemmelse af blodsukker, insulin og C-peptid med korte intervaller de næste 3 timer.

Desuden måles forsøgsdeltagernes kropssammensætning, herunder mavefedme og leverfedt, ved såkaldte DEXA- og MR-scanninger. Herudover testes forsøgsdeltagernes hjerterytmevariabilitet.

For at undersøge mulige virkningsmekanismer udtages før og efter kostperioden prøver af fedtvæv. Under lokalbedøvelse udtages med kanyle en lille prøve fedtvæv fra underhuden på maven til bestemmelse af fedtsyresammensætningen samt indhold af signalstoffer.

Alle forsøgsdeltagere deltager efter kostperioden i et 12-ugers vægttabsprogram med i alt 6 diætistkonsultationer, hvorefter alle deltagerne indkaldes til fornyet måling bl.a. for at registrere varigheden af eventuelle effekter.

Etiske overvejelser

De i forsøget indgående kostændringer svarer til, hvad der almindeligt forekommer i vestlige befolkninger med hensyn til fødevalg. De i forsøget eventuelle forårsagede ændringer i effektmål (fedtstoffer i blodet, insulinfølsomhed og kropssammensætning) under kostperioden i såvel positiv som negativ retning er forbigående og vil, efter vor bedste overbevisning, ikke medføre nogen ændring i deltagernes senere risiko for at udvikle hjertesygdom eller type 2 diabetes. Desuden har det efterfølgende vægttabsprogram potentiale til varigt at ændre forsøgspersonernes kost- og livsstilsvaner i en helbredsmæssig gunstig retning.

De i forsøget indgående undersøgelser: Effektmål, blodprøver samt blodtryk frembyder ingen målelig risiko.

Økonomisk støtte

Studiet har modtaget kr. 2.072.000 i støtte fra Det Strategiske Forskningsråd, Programkomitéen for Fødevarer og Sundhed (FøSu), kr. 58.000 fra Diabetesforeningen, og vil desuden blive søgt støttet fra offentlige og private fonde.

Perspektiv

Det gennemsnitlige daglige indtag af transfedtsyrer i Danmark er lavt, idet der med lovgivning den 1/1 2004 blev indført en øvre grænse på 2% for hvor meget transfedt, der må være i fedt i fødevarer. Der er imidlertid befolkningsgrupper i andre lande, som indtager transfedtsyrer i mængder, der giver anledning til bekymring, hvis sådanne kostvaner vedligeholdes i en årrække. Det er derfor, i international fødevarepolitisk henseende, væsentligt at indsamle data, der sikrer, at kostvejledninger, og eventuelle fødevarepolitiske tiltag, hviler på et sagligt og korrekt grundlag. Resultaterne af nærværende undersøgelser vil bidrage hertil.

Appendiks 2a: Deltagerinformation

Forespørgsel om deltagelse i et biomedicinsk forskningsprojekt med titlen:

Effekt af transfedtsyrer på risiko for udvikling af mavefedme, sukkersyge og hjertesygdom hos moderat overvægtige kvinder.

”COBRA”

Vi henvender os til dig for at spørge, om du vil deltage i et videnskabeligt forsøg ved Institut for Human Ernæring, Københavns Universitet. Forsøget er et såkaldt biomedicinsk forskningsprojekt og dets videnskabelige titel er ”Et dobbeltblindet, randomiseret kostinterventionsforsøg, der undersøger effekten af et højt indtag af transfedtsyrer på abdominal fedme samt risikomarkører for udvikling af type 2 diabetes, og hjertekarsygdom”. Projektet er initieret af Institutleder Arne Astrup.

I det følgende er en beskrivelse af selve forsøget og hvad det indebærer, hvis du beslutter dig for at deltage. Det er helt frivilligt, om du vil deltage i projektet. Selvom du beslutter, at du gerne vil deltage, kan du altid vælge at træde ud af forsøget, uden at du vil blive afkrævet en forklaring. Det gælder også, selvom du har skrevet under på, at du vil deltage.

Tag dig god tid til at læse deltagerinformationen, før du beslutter, om du vil deltage eller ej.

Baggrund

En række undersøgelser indenfor de senere år har vist, at et højt indtag af transfedtsyrer, der blandt andet findes i industrielt produceret, delvis hærdet planteolie, og som blandt andet bruges til kager, øger risikoen for udvikling af hjertesygdom. En del af denne effekt kan udspille sig gennem øget abdominal fedme (det fedt, der sidder på maven) nedsat insulinfølsomhed samt via traditionelle og nye risikofaktorer for hjertesygdom.

Hvad er formålet med forsøget?

Med nærværende undersøgelse ønsker vi at belyse effekten af en kost med et højt indhold af industrielt fremstillede transfedtsyrer sammenlignet med en transfedtsyre-fri kost på mavefedme samt en række risikomarkører for udvikling af sukkersyge og hjertesygdom. Studiet vil desuden kunne afdække, hvordan en sådan effekt kan forklares. Specielt ønsker vi at undersøge:

- om kropsvægt, kropssammensætning, sukkerstofskifte og insulinfølsomhed samt blodets indhold af fedt og kolesterol påvirkes af et øget indtag af transfedtsyrer.
- i hvilken udstrækning transfedtsyrer fra kosten aflejres i fedtvæv, og hvordan dette kan påvirke ovenstående faktorer.
- om et højt indtag af transfedtsyrer påvirker mængden af fedt i leveren, i musklerne og omkring organerne, hjerterytmen samt betændelsesreaktioner.

Desuden ønsker vi at undersøge, hvad der sker, når man går fra en kost med et højt indhold af transfedt til en normal sund kost.

Hvilken mulig nytte er der ved forsøget?

Transfedtsyrer indgår i daglig kost i bagværk og fast food produkter. Det er væsentligt for kostvejledningen af den danske befolkning, at der er viden om de nævnte kostkomponenters eventuelle heldige eller uheldige indflydelse på helbredet på langt sigt.

Det gennemsnitlige daglige indtag af transfedtsyrer i Danmark er lavt, idet der med lovgivning den 1/1 2004 blev indført en øvre grænse på 2% for hvor meget transfedt, der må være i fedt i fødevarer. Der er imidlertid befolkningsgrupper i andre lande, som indtager transfedtsyrer i mængder, der giver anledning til bekymring, hvis sådanne kostvaner vedligeholdes i en årrække. Den meget restriktive danske lovgivning er desuden under pres fra EU pga. den mulige handelshindring, som loven medfører. Det er derfor, i international fødevarepolitisk henseende, væsentligt at indsamle data, der sikrer, at kostvejledninger, og eventuelle fødevarepolitiske tiltag, hviler på et sagligt og korrekt grundlag. Information om effekten af indtag af transfedtsyrer, som kan fremkomme af dette studie, kan hjælpe de fremtidige offentlige anbefalinger og politikken inden for regulering af tilgængelighed af transfedtsyrer i fødevarer globalt. En sådan regulering vil potentielt kunne forebygge udvikling af sukkersyge og hjertesygdom i verdens befolkning.

For dig personligt vil de nævnte kostændringer, efter vort bedste skøn, ikke have positiv eller negativ indflydelse på helbredet indtaget i en så kort periode som i dette studie (16 uger). Ved deltagelse i studiet vil du modtage monitoreringer af adskillige sundhedsrelaterede faktorer, herunder BMI, blodlipider, blodtryk og kropssammensætning. Desuden vil du modtage 6 diætistkonsultationer som led i et 12-ugers vægttabsprogram. Dette vægttabsprogram har potentiale til varigt at ændre dine kost- og livsstilsvaner i en helbredsmæssig gunstig retning.

Hvad indebærer det at deltage i forsøget?

Vi udfører et koststudie, hvor ca. 60 raske moderat overvægtige postmenopausale kvinder tilfældigt inddeles i 2 lige store grupper, som i 16 uger skal følge én af følgende kosttyper:

1) En kost med et højt indhold af industrielt fremstillet transfedt (15 g transfedt per dag)
2) En transfedtfri kontrolkost med et højt indhold af oliesyre

Du vil få udleveret fødevarer, som indeholder én af de to typer fedt. Disse fødevarer (primært brød) skal du spise hver dag i 16 uger, og de skal erstatte fødevarer fra din vanlige kost. Dette vil du blive rådgivet om.

Hvis du vælger at deltage i forsøget, vil du skulle møde til besøg på Institut for Human Ernæring i alt 12 gange. Det første besøg ”screeningen” vil ligge ca. 1-12 uger før kostperiodens start, og her vil din egnethed til at indgå i studiet blive vurderet. De næste besøg ligger i starten af og hhv. 4, 8, 12, og 16 uger efter starten af kostperioden.

Efter de 16 uger, hvor du modtager forsøgskost, vil du deltage i et 12 ugers intensivt vægttabsforløb under vejledning af professionelle diætister fra Institut for Human Ernæring. I den forbindelse vil du skulle møde op til 5 separate diætistkonsultationer. Endelig vil du efter de 3 måneders vægttabsforløb skulle møde til et afsluttende besøg, en såkaldt efterundersøgelse, hvor vi vil registrere, hvor hurtigt du vender tilbage til din vanlige stand.

Der vil i forsøget blive foretaget en række undersøgelser, som vist i diagrammet på næste side. Ved hvert besøg vil der blive foretaget måling af vægt og taljeomkreds. Fem gange vil der blive taget blodprøver og ligeledes 5 gange vil du få målt dit blodtryk. I forsøget er der tre større besøg; henholdsvis umiddelbart før og efter kostperioden samt efterundersøgelsen. Disse tre besøg vil hver strække sig over 2 dage. Ved de tre store besøg vil der, efter lokalbedøvelse, med en nål blive udtaget små prøver af underhudens fedt (fedtvævsbiopsi). Desuden vil kroppens sammensætning, og dermed fedtfordelingen, blive målt ved scanning i en såkaldt DEXA-scanner. Mængden af fedt i leveren, i musklerne og omkring de indre organer vil blive bestemt ved en såkaldt MR-scanning. Endelig foretages en undersøgelse af sukkerstofskiftet efter indtagelse af 75 g druesukker. I forsøget vil vi desuden måle din hjerterymte, hvilket indebærer at du 3 gange

i forsøgets forløb i et døgn bærer en optager, som registrerer hjerterytmen. De enkelte målinger er nærmere beskrevet i det følgende:

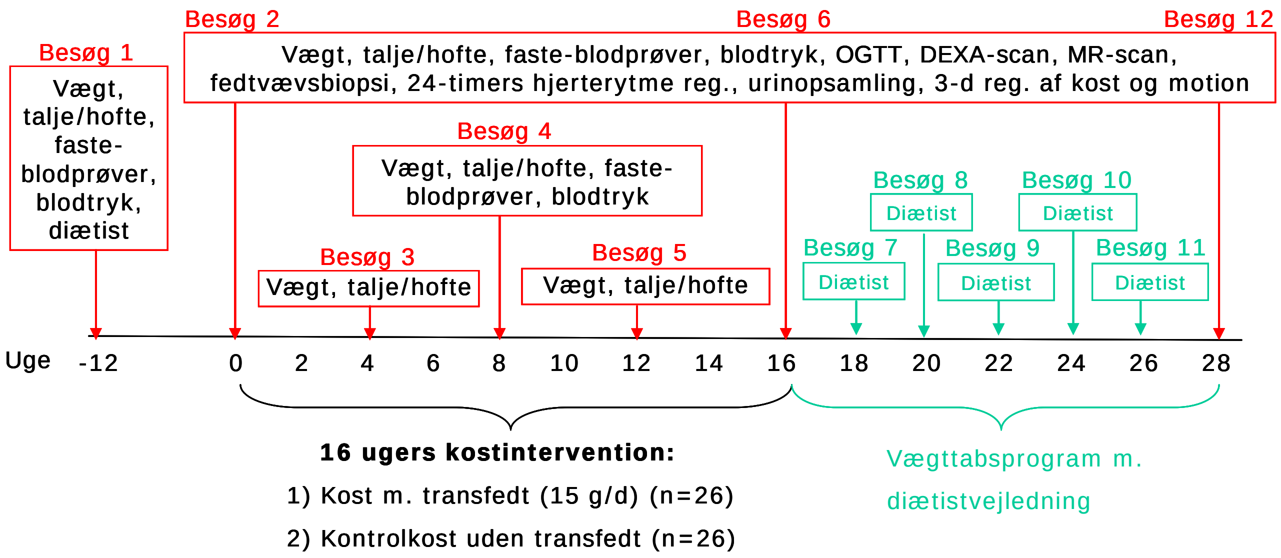

Blodprøver

Ved fem besøg vil du få taget blodprøver. Disse prøver analyseres på et senere tidspunkt, og giver os værdifulde informationer om forsøgskostens effekt på ændringer i blodets koncentration af sukker, fedtstoffer, og forskellige signalstoffer, og vil samtidig blive brugt til at analysere for sikkerhedsrelevante prøver. Til tider kan der opstå lidt lokal blødning, irritation eller infektion i forbindelse med blodprøverne. Personalet er dog veluddannede og kompetente, og risikoen for gener udover smerten ved selve stikket er derfor minimale.

Sukkerbelastning

Tre gange i løbet af forsøget vil du få foretaget sukkerbelastning. Dette involverer, at du drikker 300 milliliter vand iblandet 75 gram glukose (druesukker). Herpå måler vi din blodsukkerstigning de første tre timer efter indtagelse af sukkervandet via i alt 13 blodprøver. Til blodprøvetagningen anvender vi et såkaldt venflon, som er en meget tynd plastikkanyle, der stikkes ind i en vene på din arm og ligger der i godt 3 timer. Fordelen ved at bruge venflon er, at du kun behøver at blive stukket én gang. Da venflonen er en smule større end en almindelig nål, kan der være en smule gener i form af lettere blodudtrædning, hævelse, lokal irritation eller smerte i det berørte område. Anvendelsen af meget trænet personale begrænser dog ubehaget til et absolut minimum.

Fedtvævsprøve

Som nævnt vil vi tage tre fedtprøver i løbet af studiet. Disse vil give os oplysninger om dine fedtcellers virkemåde og sammensætningen af fedtet. Selve fedtudtagningen, der omfatter udtagningen af en lille fedtprøve fra mavens underhudsfedt med en nål, foretages under lokalbedøvelse, så du ikke føler nogen eller i værste fald meget lidt smerte. Der vil maksimalt blive taget 2 gram fedt pr. gang, og typisk ikke mere end 1gram.

MR scanning

Ved en MR-scanning (en slags røntgenundersøgelse) kan vi bestemme mængden af fedt omkring de indre organer, i musklerne samt i leveren. MR-scanningerne tager ca. 30-60 minutter og vil blive foretaget tre gange i løbet af studiet af veluddannet personale på Herlev Hospital. Teknikken til MR-scanning er baseret på anvendelse af magnetfelter og radiobølger. Derfor bliver du ikke udsat for røntgenstråler eller andre former for mulig skadelig stråling og MR-scanning er ikke forbundet med nogen form for fare eller bivirkninger. Undersøgelsen er smertefri og kan ikke mærkes på kroppen. Kun bankende lyde høres fra

maskinen, når den arbejder. Under scanningen ligger man inde i en stor cylinderformet magnet, og derfor er det vigtigt ikke at bære smykker eller andre metalgenstande under undersøgelsen. Ligeledes er det vigtigt at du fortæller, hvis du har metaldele i kroppen såsom kunstige hofteled, skruer i knogler, kirurgiske clips, eller hvis du bærer elektriske apparater såsom høreapparat eller pacemaker.

DEXA scanning

Tre gange i løbet af studiet vil vi måle din kropssammensætning ved en såkaldt DEXA-scanning. Scanneren måler kroppens sammensætning af fedt, muskler og knogler. Det er vigtigt, at du ikke har spist forud for scanningen, da maveindholdet kan påvirke resultatet af scanningen. Du vil blive scannet iført let tøj og pakket ind i et stofklæde, der lukkes med velcrobånd, så du ligger roligt i samme stilling under hele scanningen. Du skal ligge ned under scanningen, der kun tager ca. 10 min. Det eneste ubehag, der kan opstå, er i forbindelse med at skulle ligge på ryggen på et fladt leje. Scanneren måler ved hjælp af røntgenstråler i en lav dosis. Den røntgenstråling, du får ved skanningen, er meget lav og svarer omtrent til den stråling, du udsættes for ved en flyvetur fra København til Århus. Til en sammenligning giver et almindeligt røntgenbillede af hjerte og lunger en strålemængde, der er ca. 100 gange større. I forbindelse med scanningen vil vi måle din "mavehøjde".

Hjerterytmevariabilitet

Du vil tre gange i løbet af studiet få målt, hvor meget din hjerterytme varierer i løbet af ét døgn. Dette foregår ved, at du møder om morgenen, hvor fire elektroder påsættes din brystkasse og tilsluttes en optager, som bæres i et mavebælte (på størrelse med en walkman). Optageren forsynes med et digitalt hukommelseskort, hvorpå hjertets elektriske impulser gennem 24 timer optages. Du bærer optageren i 24 timer og møder næste morgen, hvor optageren afmonteres.

24-timers urinopsamling

I løbet af forsøget skal du tre gange lave en 24 timers urinopsamling. Du får til formålet udleveret en plastbeholder, som du lader vandet i, hver gang du skal på WC. Parallelt med opsamlingen får du tabletter der indeholder para-amino benzoesyre (PABA), som skal indtages tre gange dagligt (i alt 240 mg pr. urinopsamlingsdag). PABA anvendes som konserveringsmiddel i forskellige fødevarer og som beskyttende faktor i forskellige former for solcreme. Nogle mennesker er allergiske overfor PABA i solcremen. Hvis du er bekendt med at lide af en sådan allergi, bedes du orientere os herom. Det er dokumenteret at meget høje doser PABA (12-48 g pr. dag) kan føre til kræft i leveren. Stoffet har dog været anvendt i kostundersøgelser de seneste 20 år og et indtag på 240 mg pr. dag i op til 21 dage vurderes på baggrund af undersøgelser at være risikofrit.

Registrering af kost og motion

3 gange i løbet af forsøget vil vi bede dig registrere alt hvad du spiser og drikker i 3 på hinanden følgende dage. Til dette formål vil du få udleveret en digital køkkenvægt. Ligeledes 3 gange vil vi bede dig registrere din fysiske aktivitet i 3 dage.

Bivirkninger og medicin:

Under hele studiet vil du ved hvert besøg blive bedt om at fortælle om alle former for bivirkninger, du måtte have oplevet siden sidste møde, samt om enhver form for ændring i dit indtag af medicin - receptpligtig såvel som håndkøbsmedicin. Disse oplysninger, som skal sikre, at din deltagelse i studiet foregår trygt og sikkert, håndteres med den strengeste grad af fortrolighed. Skulle der optræde nogen form for komplikationer relateret til studiets aktiviteter, opfordres du til at orientere personalet herom hurtigst muligt.

Er der bivirkninger/risiko?

Eventuelle ubehag, der måtte forekomme blandt deltagende forsøgspersoner i dette studie, forventes i alle tilfælde at være af lettere og forbigående art. Risici ved deltagelsen er begrænset til mindre ubehag ved blodprøvetagning og foretagelsen af fedtvævsprøver, som kan medføre mindre blodsamlinger ved prøvetagningsstedet. Rent praktisk vil du som deltager have ulejligheden med møde til informationssamtale

og besøgene i studiet. Der vil være ulemper i forbindelse med bæring af de nævnte apparater til registrering af hjerterytmen. Der er efter vor bedste skøn ingen bivirkning ved brug af den transfedtsyreberigede kost, som anvendes i forsøget.

Skulle der mod forventning i løbet af forsøget opstå bivirkninger, som anses for væsentlige, vil din deltagelse i forsøget blive afbrudt.

Forsøget som helhed vil blive afbrudt, hvis der uventet skulle opstå alvorlige bivirkninger, som kan skyldes forsøgsprocedurerne.

Hvor foregår forsøget?

Forsøget vil foregå på Institut for Human Ernæring, Det Biovidenskabelige Fakultet, Københavns Universitet, Rolighedsvej 30, 1958 Frederiksberg C.

Hvem kan deltage i forsøget?

For at deltage i forsøget skal du være kvinde i alderen mellem 45 og 70 år og rask. Det skal være mere end et år siden, du havde din sidste menstruation. Du skal være moderat overvægtig, dvs. dit BMI skal ligge mellem 25-32 kg/m^2. Du kan beregne dit BMI ved at dividere din vægt (målt i kg) med din højde gange højde (målt i meter). Dit taljemål skal desuden være større end eller lig med 80 cm.

Du kan IKKE deltage, hvis du lider af følgende:

- Sukkersyge eller andre kroniske sygdomme
- Tidligere eller nuværende hjertesygdom
- Forhøjet blodtryk
- For højt indhold af kolesterol eller triglycerider i blodet
- Afhængighed af alle former for medikamenter, alkohol eller euforiserende stoffer

Du må ikke indtage nogen former for kosttilskud under forsøget eller én måned før forsøgets start. Du må ikke være elite-idrætsudøver, og du må ikke være ryger. Endelig må du ikke samtidig deltage i andre forsøg.

Hvem kan få oplysninger om projektet?

Dit navn eller identitet vil ikke fremgå eller kunne fastslås ud fra rapporter eller publikationer fra dette forsøg. Al information vedrørende din deltagelse i forsøget vil kun være tilgængelig for personer på Institut for Human Ernæring. Disse personer er underlagt tavshedspligt. Du kan selv få adgang til at se alle papirerne vedrørende selve forsøget, bortset fra de dele, der indeholder private oplysninger om de andre deltagere. Undersøgelsesresultater vil, efter ønske, blive givet til den enkelte forsøgsdeltager ved afslutning af dataindsamling og -behandling.

Resultaterne fra forsøget vil blive publiceret i videnskabelige og medicinske tidsskrifter.

Hvem har godkendt forsøget?

Forsøget er anmeldt til datatilsynet og er godkendt af De Videnskabsetiske Komitéer for Region Hovedstaden.

Hvordan er de økonomiske forhold?

Studiets økonomisk ansvarlige er Institutleder Arne Astrup. Studiet har modtaget kr. 2.072.000 i støtte fra Det Strategiske Forskningsråd, Programkomitéen for Fødevarer og Sundhed (FøSu), og kr. 58.000 fra Diabetesforeningen. Støtten udbetales til Institut for Human Ernæring. Arne Astrup har ingen økonomisk tilknytning til støttegivere. Studiet søges i øvrigt støttet af såvel offentlige som private fonde.

Der udbetales honorar på 3000 kr. (beskattet som A-indkomst) efter endt deltagelse i besøg 1-6. Yderligere udbetales honorar på 1500 kr. (beskattet som A-indkomst), såfremt besøg 7-12 gennemføres.

Frivillig deltagelse

Det er frivilligt at deltage. Du opfordres til at læse vedlagte tillæg ”Forsøgspersonens rettigheder i et biomedicinsk forskningsprojekt”. Du kan på ethvert tidspunkt og uden begrundelse trække samtykket til deltagelse tilbage og udtræde af forsøget.

Tilsvarende kan du blive udelukket fra deltagelse i forsøget, hvis det er åbenlyst, at du ikke følger studiets retningslinier.

Projektleder og kontaktperson

Har du spørgsmål, er du velkommen til at kontakte:

Daglig ansvarlig forsøgskoordinator:

Navn: Nathalie Tommerup Bendsen, PhD-studerende
Adresse: Institut for Human Ernæring, Det Biovidenskabelige Fakultet, København Universitet
Rolighedvej 30, 1958 Frederiksberg C
Telefon: 3533 3633 (dag)
Email: nathalie@life.ku.dk

Overordnet ansvarlig forsøgsleder:

Navn: Arne Astrup, Læge og Professor, Institutleder på Institut for Human Ernæring
Adresse: Institut for Human Ernæring, Det Biovidenskabelige Fakultet, København Universitet
Rolighedvej 30, 1958 Frederiksberg C
Telefon: 3533 2476
Sekretær tlf: 3533 2502

Appendiks 2b. Deltagerinformation, normalvægtige referencer

Forespørgsel om deltagelse som normalvægtig referencedeltager i et biomedicinsk forskningsprojekt med titlen:

Effekt af transfedtsyrer på risiko for udvikling af mavefedme, sukkersyge og hjertesygdom hos moderat overvægtige kvinder.
”COBRA”

Vi henvender os til dig for at spørge, om du vil deltage i et videnskabeligt forsøg ved Institut for Human Ernæring, Københavns Universitet. Forsøget er et såkaldt biomedicinsk forskningsprojekt og dets videnskabelige titel er ”Et dobbeltblindet, randomiseret kostinterventionsforsøg, der undersøger effekten af et højt indtag af transfedtsyrer på abdominal fedme samt risikomarkører for udvikling af type 2 diabetes, og hjertekarsygdom”. Projektet er initieret af Institutleder Arne Astrup.

I det følgende er en beskrivelse af selve forsøget og hvad det indebærer, hvis du beslutter dig for at deltage som normalvægtig referencedeltager. Det er helt frivilligt, om du vil deltage i projektet. Selvom du beslutter, at du gerne vil deltage, kan du altid vælge at træde ud af forsøget, uden at du vil blive afkrævet en forklaring. Det gælder også, selvom du har skrevet under på, at du vil deltage.

Tag dig god tid til at læse deltagerinformationen, før du beslutter, om du vil deltage eller ej.

Baggrund

En række undersøgelser indenfor de senere år har vist, at et højt indtag af transfedtsyrer, der blandt andet findes i industrielt produceret, delvis hærdet planteolie, og som blandt andet bruges til kager, øger risikoen for udvikling af hjertesygdom. En del af denne effekt kan udspille sig gennem øget abdominal fedme (det fedt, der sidder på maven) nedsat insulinfølsomhed samt via traditionelle og nye risikofaktorer for hjertesygdom.

Hvad er formålet med forsøget?

Forsøgets hovedstudie har til formål at belyse effekten af en kost med et højt indhold af industrielt fremstillede transfedtsyrer sammenlignet med en transfedtsyre-fri kost på mavefedme samt en række risikomarkører for udvikling af sukkersyge og hjertesygdom hos moderat overvægtige kvinder. Studiet vil desuden kunne afdække, hvordan en sådan effekt kan forklares. Specielt ønsker vi at undersøge:

- om kropsvægt, kropssammensætning, sukkerstofskifte og insulinfølsomhed samt blodets indhold af fedt og kolesterol påvirkes af et øget indtag af transfedtsyrer.
- i hvilken udstrækning transfedtsyrer fra kosten aflejres i fedtvæv, og hvordan dette kan påvirke ovenstående faktorer.
- om et højt indtag af transfedtsyrer påvirker mængden af fedt i leveren, i musklerne og omkring organerne, hjerterytmen samt betændelsesreaktioner.

Vi ønsker at foretage samme målinger på en gruppe normalvægtige referencepersoner, som ikke skal deltage i koststudiet. Det spørger vi om din hjælp til. Formålet hermed er, at etablere et mål for, hvor forskellige hovedstudiets overvægtige deltagere er fra en normalvægtig referencegruppe mht. de undersøgte parametre.

Hvilken mulig nytte er der ved forsøget?

Transfedtsyrer indgår i daglig kost i bagværk og fast food produkter. Det er væsentligt for kostvejledningen af den danske befolkning, at der er viden om de nævnte kostkomponenters eventuelle heldige eller uheldige indflydelse på helbredet på langt sigt.

Det gennemsnitlige daglige indtag af transfedtsyrer i Danmark er lavt, idet der med lovgivning den 1/1 2004 blev indført en øvre grænse på 2% for hvor meget transfedt, der må være i fedt i fødevarer. Der er imidlertid befolkningsgrupper i andre lande, som indtager transfedtsyrer i mængder, der giver anledning til bekymring, hvis sådanne kostvaner vedligeholdes i en årrække. Den meget restriktive danske lovgivning er desuden under pres fra EU pga. den mulige handelshindring som loven medfører. Det er derfor, i international fødevarepolitisk henseende, væsentligt at indsamle data, der sikrer, at kostvejledninger, og eventuelle fødevarepolitiske tiltag, hviler på et sagligt og korrekt grundlag. Information om effekten af indtag af transfedtsyrer, som kan fremkomme af dette studie, kan hjælpe de fremtidige offentlige anbefalinger og politikken inden for regulering af tilgængelighed af transfedtsyrer i fødevarer globalt. En sådan regulering vil potentielt kunne forebygge udvikling af sukkersyge og hjertesygdom i verdens befolkning.

Din deltagelse i studiet som normalvægtig referenceperson vil gøre det mulig for os at måle i hvilken grad forsøgets overvægtige forsøgspersoner har 1) nedsat sukkerstofskifte, 2) ændringer i fedtet, der sidder på maven, 3) fejlregulering af signalstoffer i blodbanen, 4) ændringer i hjerterytmen, 5)øget mængde fedt i leveren, omkring organerne og i musklerne (målt med MR-scanner). Disse ændringer hos overvægtige er vigtige at få påvist. Det vil øge forståelse af i hvor høj grad transfedt i kosten vil påvirke disse variable i forhold til et reference-niveau eller normalt niveau.

Ved deltagelse i studiet vil du modtage monitoreringer af adskillige sundhedsrelaterede faktorer, herunder BMI, blodlipider, blodtryk og kropssammensætning.

Hvad indebærer det at deltage i forsøget?

I hovedstudiet vil ca. 60 raske moderat overvægtige kvinder tilfældigt inddeles i 2 lige store grupper, som i 16 uger skal følge én af følgende kosttyper:

1) En kost med et højt indhold af industrielt fremstillet transfedt (15 g transfedt per dag)

2) En transfedtfri kontrolkost med et højt indhold af oliesyre

Som referenceperson skal du ikke indtage forsøgskost. Du skal blot møde op til 2 besøg på Institut for Human ernæring. Ved det første besøg ”screeningen” vil din egnethed til at indgå i studiet blive vurderet. Ca. 1-12 uger herefter deltager du i et undersøgelsesbesøg, som modsvarer det første besøg for de overvægtige forsøgspersoner, der deltager i kostinterventionen.

Undersøgelsesbesøget vil strække sig over 2 dage, hvor der vil blive foretaget en række undersøgelser. Du vil få målt din vægt og talje- og hofteomkreds. Du vil få taget blodprøver og få målt dit blodtryk. Efter lokalbedøvelse vil der med en nål blive udtaget små prøver af underhudens fedt (fedtvævsbiopsi). Ligeledes vil kroppens sammensætning, og dermed fedtfordelingen, blive målt ved scanning i en såkaldt DEXA-scanner. Mængden af fedt i leveren, i musklerne og omkring de indre organer vil blive bestemt ved en såkaldt MR-scanning. Endelig foretages en undersøgelse af sukkerstofskiftet efter indtagelse af 75 g druesukker. I forsøget vil vi desuden måle din hjerterymte, hvilket indebærer at du i et døgn bærer en optager, som registrerer hjerterytmen. De enkelte målinger er nærmere beskrevet i det følgende:

Blodprøver

Blodprøverne giver os information om blodets koncentration af sukker, fedtstoffer, og forskellige signalstoffer, og vil samtidig blive brugt til at analysere for sikkerhedsrelevante prøver. Til tider kan der opstå lidt lokal blødning, irritation eller infektion i forbindelse med blodprøverne. Personalet er dog veluddannede og kompetente, og risikoen for gener udover smerten ved selve stikket er derfor minimale.

Sukkerbelastning

Du vil få foretaget sukkerbelastning. Dette involverer, at du drikker 300 milliliter vand iblandet 75 gram glukose (druesukker). Herpå måler vi din blodsukkerstigning de første tre timer efter indtagelse af sukkervandet via i alt 13 blodprøver. Til blodprøvetagningen anvender vi et såkaldt venflon, som er en meget tynd plastikkanyle, der stikkes ind i en vene på din arm og ligger der i godt 3 timer. Fordelen ved at bruge venflon er, at du kun behøver at blive stukket én gang. Da venflonen er en smule større end en almindelig nål, kan der være en smule gener i form af lettere blodudtrædning, hævelse, lokal irritation eller smerte i det berørte område. Anvendelsen af meget trænet personale begrænser dog ubehaget til et absolut minimum.

Fedtvævsprøve

Som nævnt vil vi tage en fedtvævsprøve. Denne vil give os oplysninger om dine fedtcellers virkemåde og sammensætningen af fedtet. Selve fedtudtagningen, der omfatter udtagningen af en lille fedtprøve fra mavens underhudsfedt med en nål, foretages under lokalbedøvelse, så du ikke føler nogen eller i værste fald meget lidt smerte. Der vil maksimalt blive taget 2 gram fedt, og typisk ikke mere end 1 gram.

MR scanning

Ved en MR-scanning (en slags røntgenundersøgelse) kan vi bestemme mængden af fedt omkring de indre organer, i leveren samt i musklerne. MR-scanningen tager ca. 30-60 minutter og vil blive foretaget af veluddannet personale på Herlev Hospital. Teknikken til MR-scanning er baseret på anvendelse af magnetfelter og radiobølger. Derfor bliver du ikke udsat for røntgenstråler eller andre former for mulig skadelig stråling og MR-scanning er ikke forbundet med nogen form for fare eller bivirkninger. Undersøgelsen er smertefri og kan ikke mærkes på kroppen. Kun bankende lyde høres fra maskinen, når den arbejder. Under scanningen ligger man inde i en stor cylinderformet magnet, og derfor er det vigtigt ikke at bære smykker eller andre metalgenstande under undersøgelsen. Ligeledes er det vigtigt at du fortæller, hvis du har metaldele i kroppen såsom kunstige hofteled, skruer i knogler, kirurgiske clips, eller hvis du bærer elektriske apparater såsom høreapparat eller pacemaker.

DEXA scanning

Vi vil måle din kropssammensætning ved en såkaldt DEXA-scanning. Scanneren måler kroppens sammensætning af fedt, muskler og knogler. Det er vigtigt, at du ikke har spist forud for scanningen, da maveindholdet kan påvirke resultatet af scanningen. Du vil blive scannet iført let tøj og pakket ind i et stofklæde, der lukkes med velcrobånd, så du ligger roligt i samme stilling under hele scanningen. Du skal ligge ned under scanningen, der kun tager ca. 10 min. Det eneste ubehag, der kan opstå, er i forbindelse med at skulle ligge på ryggen på et fladt leje. Scanneren måler ved hjælp af røntgenstråler i en lav dosis. Den røntgenstråling, du får ved skanningen, er meget lav og svarer omtrent til den stråling, du udsættes for ved en flyvetur fra København til Århus. Til en sammenligning giver et almindeligt røntgenbillede af hjerte og lunger en strålemængde, der er ca. 100 gange større. I forbindelse med scanningen vil vi måle din "mavehøjde".

Hjerterytmevariabilitet

Vi vil også måle, hvor meget din hjerterytme varierer i løbet af et døgn. Dette foregår ved, at du møder om morgenen, hvor fire elektroder påsættes din brystkasse og tilsluttes en optager, som bæres i et mavebælte (på størrelse med en walkman). Optageren forsynes med et digitalt hukommelseskort, hvorpå hjertets elektriske impulser gennem 24 timer optages. Du bærer optageren i 24 timer og møder næste morgen, hvor optageren afmonteres.

24-timers urinopsamling

Vi vil også bede dig lave en 24 timers urinopsamling. Du får til formålet udleveret en plastbeholder, som du lader vandet i, hver gang du skal på WC. Parallelt med opsamlingen får du tabletter der indeholder para-amino benzoesyre (PABA), som skal indtages tre gange dagligt (i alt 240 mg pr. urinopsamlingsdag). PABA anvendes som konserveringsmiddel i forskellige fødevarer og som beskyttende faktor i forskellige former for solcreme. Nogle mennesker er allergiske overfor PABA i solcremen. Hvis du er bekendt med at lide af en

sådan allergi, bedes du orientere os herom. Det er dokumenteret at meget høje doser PABA (12-48 g pr. dag) kan føre til kræft i leveren. Stoffet har dog været anvendt i kostundersøgelser de seneste 20 år og et indtag på 240 mg pr. dag i op til 21 dage vurderes på baggrund af undersøgelser at være risikofrit.

Registrering af kost og motion

Du skal registrere alt hvad du spiser og drikker i 3 på hinanden følgende dage. Til dette formål vil du få udleveret en digital køkkenvægt. Ligeledes skal du registrere din fysiske aktivitet i 3 dage.

Bivirkninger og medicin:

Du vil ved undersøgelsesbesøget blive bedt om at fortælle om alle former for bivirkninger, du måtte have oplevet siden første møde, samt om enhver form for ændring i dit indtag af medicin - receptpligtig såvel som håndkøbsmedicin. Disse oplysninger, som skal sikre, at din deltagelse i studiet foregår trygt og sikkert, håndteres med den strengeste grad af fortrolighed. Skulle der optræde nogen form for komplikationer relateret til studiets aktiviteter, opfordres du til at orientere personalet herom hurtigst muligt.

Er der bivirkninger/risiko?

Eventuelle ubehag, der måtte forekomme blandt deltagende forsøgspersoner i dette studie, forventes i alle tilfælde at være af lettere og forbigående art. Risici ved deltagelsen er begrænset til mindre ubehag ved blodprøvetagning og foretagelsen af fedtprøver, som kan medføre mindre blodsamlinger ved prøvetagningsstedet. Rent praktisk vil du som deltager have ulejligheden med møde til informationssamtale og de to besøg i studiet. Der vil være ulemper i forbindelse med bæring af de nævnte apparater til registrering af hjerterytmen. Skulle der mod forventning i løbet af forsøget opstå bivirkninger, som anses for væsentlige, vil din deltagelse i forsøget blive afbrudt. Forsøget som helhed vil blive afbrudt, hvis der uventet skulle opstå alvorlige bivirkninger, som kan skyldes forsøgsprocedurerne.

Hvor foregår forsøget?

Forsøget vil foregå på Institut for Human Ernæring, Det Biovidenskabelige Fakultet, Københavns Universitet, Rolighedsvej 30, 1958 Frederiksberg C.

Hvem kan deltage i forsøget?

For at deltage i forsøget som referencedeltager skal du være kvinde i alderen mellem 45 og 70 år og rask. Det skal være mere end et år siden, du havde din sidste menstruation. Du skal være normalvægtig i det nedre normalområde, dvs. dit BMI skal ligge mellem 18-23 kg/m^2. Du kan beregne dit BMI ved at dividere din vægt (målt i kg) med din højde gange højde (målt i meter). Dit taljemål skal desuden være mindre end 80 cm.

Du kan IKKE deltage, hvis du lider af følgende:

- Sukkersyge eller andre kroniske sygdomme
- Tidligere eller nuværende hjertesygdom
- Forhøjet blodtryk
- For højt indhold af kolesterol eller triglycerider i blodet
- Afhængighed af alle former for medikamenter, alkohol eller euforiserende stoffer

Du må ikke indtage nogen former for kosttilskud under forsøget eller én måned før forsøgets start. Du må ikke være elite-idrætsudøver, og du må ikke være ryger. Endelige må du ikke samtidig deltage i andre forsøg.

Hvem kan få oplysninger om projektet?

Dit navn eller identitet vil ikke fremgå eller kunne fastslås ud fra rapporter eller publikationer fra dette forsøg. Al information vedrørende din deltagelse i forsøget vil kun være tilgængelig for personer på Institut for Human Ernæring. Disse personer er underlagt tavshedspligt. Du kan selv få adgang til at se alle papirerne

vedrørende selve forsøget, bortset fra de dele, der indeholder private oplysninger om de andre deltagere. Undersøgelsesresultater vil, efter ønske, blive givet til den enkelte forsøgsdeltager ved afslutning af dataindsamling og -behandling.

Resultaterne fra forsøget vil blive publiceret i videnskabelige og medicinske tidsskrifter.

Hvem har godkendt forsøget?

Forsøget er anmeldt til datatilsynet og er godkendt af De Videnskabsetiske Komitéer for Region Hovedstaden.

Hvordan er de økonomiske forhold?

Studiets økonomisk ansvarlige er Institutleder Arne Astrup. Studiet har modtaget kr. 2.072.000 i støtte fra Det Strategiske Forskningsråd, Programkomitéen for Fødevarer og Sundhed (FøSu), og kr. 58.000 fra Diabetesforeningen. Støtten udbetales til Institut for Human Ernæring. Arne Astrup har ingen økonomisk tilknytning til støttegivere. Studiet søges i øvrigt støttet af såvel offentlige som private fonde.

Der udbetales honorar på 2500 kr. (beskattet som A-indkomst) til forsøgsdeltagere i referencegruppen, når alle undersøgelser er gennemført.

Frivillig deltagelse

Det er frivilligt at deltage. Du opfordres til at læse vedlagte tillæg "Forsøgspersonens rettigheder i et biomedicinsk forskningsprojekt". Du kan på ethvert tidspunkt og uden begrundelse trække samtykket til deltagelse tilbage og udtræde af forsøget.

Tilsvarende kan du blive udelukket fra deltagelse i forsøget, hvis det er åbenlyst, at du ikke følger studiets retningslinier.

Projektleder og kontaktperson

Har du spørgsmål, er du velkommen til at kontakte:

Daglig ansvarlig forsøgskoordinator:

Navn:	Nathalie Tommerup Bendsen, PhD-studerende
Adresse:	Institut for Human Ernæring, Det Biovidenskabelige Fakultet, København Universitet<br>Rolighedvej 30, 1958 Frederiksberg C
Telefon:	3533 3633 (dag)
Email:	nathalie@life.ku.dk

Overordnet ansvarlig forsøgsleder:

Navn:	Arne Astrup, Læge og Professor, Institutleder på Institut for Human Ernæring
Adresse:	Institut for Human Ernæring, Det Biovidenskabelige Fakultet, København Universitet<br>Rolighedvej 30, 1958 Frederiksberg C
Telefon:	3533 2476
Sekretær tlf:	3533 2502

Appendiks 3: "Dine rettigheder som forsøgsperson i et biomedicinsk forskningsprojekt"

DEN CENTRALE VIDENSKABSETISKE KOMITÉ

The Danish National Committee on Biomedical Research Ethics

Forsøgspersonens rettigheder i et biomedicinsk forskningsprojekt.

Som deltager i et biomedicinsk forskningsprojekt skal du vide at:

- din deltagelse i forskningsprojektet er helt frivillig og kan kun ske efter, at du har fået både skriftlig og mundtlig information om forskningsprojektet og underskrevet samtykkeerklæringen
- du til enhver tid mundtligt, skriftligt eller ved anden klar tilkendegivelse kan trække dit samtykke til deltagelse tilbage og udtræde af forskningsprojektet. Såfremt du trækker dit samtykke tilbage påvirker dette ikke din ret til nuværende eller fremtidig behandling eller andre rettigheder, som du måtte have
- du har ret til at tage et familiemedlem, en ven eller en bekendt med til informationssamtalen
- du har ret til betænkningstid, før du underskriver samtykkeerklæringen
- oplysninger om dine helbredsforhold, øvrige rent private forhold og andre fortrolige oplysninger om dig, som fremkommer i forbindelse med forskningsprojektet, er omfattet af tavshedspligt
- opbevaring af oplysninger om dig, herunder oplysninger i dine blodprøver og væv, sker efter reglerne i lov om behandling af personoplysninger og sundhedsloven
- der er mulighed for at få aktindsigt i forsøgsprotokoller efter offentlighedslovens bestemmelser. Det vil sige, at du kan få adgang til at se alle papirer vedrørende din deltagelse i forsøget, bortset fra de dele, som indeholder forretningshemmeligheder eller fortrolige oplysninger om andre
- der er mulighed for at klage og få erstatning efter reglerne i lov om klage- og erstatningsadgang inden for sundhedsvæsenet

(Dette tillæg udgives af Den Centrale Videnskabsetiske komité og kan vedhæftes den skriftlige information om det biomedicinske forskningsprojekt. Spørgsmål til et projekt skal rettes til den regionale komité, som har godkendt projektet)

Appendiks 4: Procedure for afgivelse af mundtlig deltagerinformation

”COBRA”

Personer, der reflekterer positivt på annoncen til hvervning af forsøgsdeltagere, vil efter ønske få fremsendt deltagerinformationen, hvori det er angivet, at der forespørges om deltagere til et biomedicinsk forskningsprojekt, enten via e-mail eller almindelig post.

Desuden inviteres personen til at deltage i et tidsfastsat ca. halvanden time varende informationsmøde på Institut for Human Ernæring. Personen oplyses forinden om retten til at medbringe en bisidder ved mødet, samt om at man ikke behøver at tage stilling til evt. deltagelse i forskningsprojektet ved selve mødet, men at samtykke kan afgives op til en uge efter informationsmødets afholdelse.

Mødet afholdes af den ansvarlige for det praktiske forløb af studiet, PhD-studerende Nathalie Tommerup Bendsen.

Mødet indledes med et kort foredrag, der i lægmandstermer beskriver den videnskabelige baggrund for forsøget.

Der redegøres for vilkår for forsøgsdeltagelse, med understregning af det frivillige aspekt.

Herefter gennemgås forsøgets praktiske forløb samt risiko for bivirkninger i detaljer. Det understreges, at forsøgshonoraret er skattepligtigt. Der vil til slut være tid til at stille spørgsmål.

Skriftlig deltagerinformation og samtykkeerklæring uddeles sammen med ”Dine rettigheder som forsøgsperson”. På samtykkeerklæringen skal deltageren markere, om vedkommende ønsker at få væsentlige oplysninger om egen helbredstilstand, der måtte fremkomme under gennemførelse af projektet, samt om der ønskes information om de i projektet opnåede resultater efter projektets afslutnings.

Deltagerne kan vælge at underskrive samtykkeerklæringen efter mødet, sammen med den studieansvarlige. Alternativt kan deltageren medtage deltagerinformation, i hvilket tilfælde deltageren bedes tage stilling til samtykke i løbet af førstkommende uge. Allersenest ved screening skal samtykkeerklæringen underskrives.

Appendiks 5: Samtykkeerklæring

Samtykkeerklæring

"COBRA - Effekt af transfedtsyrer på risiko for udvikling af mavefedme, type-2 diabetes og hjertesygdom hos moderat overvægtige kvinder"

Erklæring fra den forsøgsansvarlige:

Jeg erklærer, at nedenstående forsøgsdeltager har modtaget mundtlig og skriftlig information om forskningsprojektet. Efter min overbevisning er der givet tilstrækkelig information, herunder om fordele og ulemper, til at træffe et informeret valg.

Forsøgsansvarliges navn

Dato

Underskrift

Erklæring fra forsøgsdeltageren:

Jeg bekræfter hermed:

- at jeg har modtaget og forstået, såvel skriftlig som mundtlig, information om forskningsprojektet.

- at jeg ønsker at deltage i forskningsprojektet.

- at jeg er informeret om, at min deltagelse er frivillig, og at jeg når som helst kan trække mit tilsagn om deltagelse tilbage og udtræde af forsøget.

- at jeg er indforstået med, at der udtages biologisk materiale fra mig, og at dette materiale opbevares i en forsøgsbiobank.

Jeg ønsker at få væsentlige oplysninger om min helbredstilstand, der måtte fremkomme under gennemførelse af projektet……………………………………………………………Ja ☐ Nej ☐

Jeg ønsker information om de i projektet opnåede resultater efter projektets afslutning…….. Ja ☐ Nej ☐

Forsøgsdeltagerens navn

Dato

Underskrift

Telefon

E-mail

Kopi til forsøgsdeltager

Samtykkeerklæring

”COBRA - Effekt af transfedtsyrer på risiko for udvikling af mavefedme, type-2 diabetes og hjertesygdom hos moderat overvægtige kvinder”

Erklæring fra den forsøgsansvarlige:

Jeg erklærer, at nedenstående forsøgsdeltager har modtaget mundtlig og skriftlig information om forskningsprojektet. Efter min overbevisning er der givet tilstrækkelig information, herunder om fordele og ulemper, til at træffe et informeret valg.

Forsøgsansvarliges navn

Dato

Underskrift

Erklæring fra forsøgsdeltageren:

Jeg bekræfter hermed:

- at jeg har modtaget og forstået, såvel skriftlig som mundtlig, information om forskningsprojektet.

- at jeg ønsker at deltage i forskningsprojektet.

- at jeg er informeret om, at min deltagelse er frivillig, og at jeg når som helst kan trække mit tilsagn om deltagelse tilbage og udtræde af forsøget.

- at jeg er indforstået med, at der udtages biologisk materiale fra mig, og at dette materiale opbevares i en forsøgsbiobank.

Jeg ønsker at få væsentlige oplysninger om min helbredstilstand, der måtte fremkomme under gennemførelse af projektet……………………………………………………………….. Ja ☐ Nej ☐

Jeg ønsker information om de i projektet opnåede resultater efter projektets afslutning…….. Ja ☐ Nej ☐

Forsøgsdeltagerens navn

Dato

Underskrift

Telefon

E-mail

Kopi til forsøgsansvarlig

Appendiks 6a: Annoncetekst til hvervning af deltagere

Overvægtige kvinder søges til kostforsøg på Institut for Human Ernæring

Til et forskningsprojekt, som har til formål at undersøge effekten af et højt indtag af transfedtsyrer på udvikling af fedme og på risikomarkører for type 2 diabetes og hjertesygdom søger vi 52 moderat overvægtige postmenopausale kvinder.

For at kunne deltage skal du:

- være mellem 45 og 70 år
- være moderat overvægtig (BMI 25 - 32 kg/m^2).
- have en taljeomkreds lig med eller større end 80 cm.
- ikke have sukkersyge eller hjertesygdom
- ikke have forhøjet blodtryk, eller for højt indhold af kolesterol eller triglycerider i blodet
- ikke have haft menstruation i mere end et år
- ikke være ryger

Hvad indebærer forsøget for dig?

Du vil i en periode på 16 uger få udleveret 2 boller, indeholdende enten transfedtsyrer eller kontrolfedt, som du skal spise hver dag og som skal erstatte fødevarer fra din vanlige kost. I forsøget indgår 7 besøg på Institut for Human Ernæring samt en række diætistkonsultationer, og forsøget vil for dig i alt strække sig over ca. 32 uger.

Efter de 16 uger, hvor du modtager forsøgskost vil du deltage i et

3 måneders intensivt vægttabsforløb

under vejledning af professionelle diætister fra

Institut for Human Ernæring.

Desuden vil du blive inviteret til et foredrag om ernæring ved Institutleder Arne Astrup.

Der vil i forsøget blive foretaget en række undersøgelser. Vi vil måle din kropssammensætning, og dermed fedtfordeling ved scanning i en såkaldt DEXA-scanner. Mængden af fedt i leveren, i musklerne og omkring organerne vil blive bestemt ved en såkaldt MR-scanning. Du vil få taget blod- og fedtvævsprøver og du vil få foretaget en undersøgelse af sukkerstofskiftet. Desuden vil vi måle din hjerterytme.

Forsøget er ikke forbundet med nogen gener for forsøgspersonerne, bortset fra evt. begrænset ubehag ved udtagning af blod- og fedtvævsprøver.

Når du har gennemført første del af forsøget, vil du modtage 3000 kr. i godtgørelse (skattepligtigt). Yderligere udbetales honorar på 1500 kr. (beskattet som A-indkomst), såfremt du også gennemfører et opfølgningsbesøg efter vægttabsforløbet.

Du kan få mere information om forsøget ved at kontakte:

Forsøgskoordinator Nathalie Bendsen (PhD-studerende) på tlf. XXXX XXXX ml. kl. X og X eller via e-mail: XXX

Appendiks 6b: Annoncetekst til hvervning af normalvægtige referencer

Normalvægtige kvinder søges til forsøg på Institut for Human Ernæring

Til et forskningsprojekt, som har til formål at undersøge effekten af et højt indtag af transfedtsyrer på udvikling af fedme og på risikomarkører for type 2 diabetes og hjertesygdom søger vi 20 normalvægtige postmenopausale kvinder, som skal udgøre en referencegruppe til forsøgets overvægtige forsøgsdeltagere. Du skal derfor ikke deltage i kostinterventionen, men blot gennemgå en række undersøgelser af din sundhedstilstand.

For at kunne deltage skal du:

- være mellem 45 og 70 år
- være normalvægtig i det nedre normalområde (BMI 19-23 kg/m^2).
- have en taljeomkreds mindre end 80 cm.
- ikke have sukkersyge eller hjertesygdom
- ikke have forhøjet blodtryk, eller for højt indhold af kolesterol eller triglycerider i blodet
- ikke have haft menstruation i mere end et år
- ikke være ryger

Hvad indebærer forsøget for dig?

Du vil skulle møde op til 2 besøg på Institut for Human Ernæring. Ved begge besøg vil du blive målt og vejet og du vil få taget blodprøver. Ved besøg 2 vil vi endvidere måle din kropssammensætning, og dermed fedtfordeling ved scanning i en såkaldt DEXA-scanner. Mængden af fedt i leveren, i musklerne og omkring organerne vil blive bestemt ved en såkaldt MR-scanning. Du vil få taget en fedtvævsprøve og du vil få foretaget en undersøgelse af sukkerstofskiftet. Desuden vil vi måle din hjerterytme.

Forsøget er ikke forbundet med nogen gener for forsøgspersonerne, bortset fra evt. begrænset ubehag ved udtagning af blod- og fedtvævsprøver.

Når du har gennemført de to besøg, vil du modtage 2500 kr. i godtgørelse (skattepligtigt).

Desuden vil du blive inviteret til et foredrag om ernæring ved Institutleder Arne Astrup.

Du kan få mere information om forsøget ved at kontakte:

Forsøgskoordinator Nathalie Bendsen (PhD-studerende) på tlf. XXXX XXXX ml. kl. X og X eller via e-mail: XXX

Appendiks 7: Skema for registrering af fysisk aktivitet

Initialer:____________________Forsøgsnr.:_______________________ Dato:_____________________

Du skal skrive den aktivitetsværdi, der passer bedst for hvert kvarter af døgnets 24 timer. Aktivitetsværdierne fremgår af vedlagte vejledning. Hvis du er i tvivl om, hvilken værdi du skal skrive, bør du notere aktiviteten og spørge ved næste fremmøde.

Min / Time	0-15	15-30	30-45	45-60
0				
1				
2				
3				
4				
5				
6				
7				
8				
9				
10				
11				
12				
13				
14				
15				
16				
17				
18				
19				
20				
21				
22				
23				

Tabel over aktiviteter og den tilsvarende aktivitetsværdi

**Aktivitetsværdi**	**Eksempler på aktiviteter**
1	Sover, hviler liggende
2	Siddende aktivitet:<br>Spiser, lytter, skriver, ser tv
3	Let stående aktivitet:<br>Laver mad, vasker op, barbering
4	Går langsomt (< 4 km/t)<br>Kører bil/knallert/motorcykel<br>Påklædning, badning
5	Let arbejde:<br>Gulvfejning, vinduespudsning, maler, diverse husarbejde<br>Går i normalt tempo (4 – 6 km/t)
6	Fritidsaktiviteter:<br>Bordtennis, baseball, golf, volleyball, roning, bowling<br>Langsom cycling (<10 km/t)
7	Manuelt arbejde ved moderat tempo:<br>Løft af tunge ting, snerydning
8	Fritidsaktiviteter med højt tempo:<br>Dans, badminton, gymnastik, svømning, tennis, ridning, roning, skiløb<br>Cykling (> 15 km/t)<br>Går i hurtigt tempo (> 6 km/t)
9	Anstrengt aktivitet:<br>Badminton, svømning, tennis, langrend (> 8 km/t)<br>Løb (> 9 km/t)
